# Use of Whole-Genome Sequencing to Unravel the Genetic Diversity of a Prevalent *Mycobacterium bovis* Spoligotype in a Multi-Host Scenario in Spain

**DOI:** 10.3389/fmicb.2022.915843

**Published:** 2022-07-11

**Authors:** Pilar Pozo, Victor Lorente-Leal, Suelee Robbe-Austerman, Jessica Hicks, Tod Stuber, Javier Bezos, Lucia de Juan, Jose Luis Saez, Beatriz Romero, Julio Alvarez, on behalf of the Spanish Network on Surveillance Monitoring of Animal Tuberculosis

**Affiliations:** ^1^VISAVET Health Surveillance Centre, Universidad Complutense de Madrid, Madrid, Spain; ^2^Departamento de Sanidad Animal, Facultad de Veterinaria, Universidad Complutense de Madrid, Madrid, Spain; ^3^National Veterinary Services Laboratories, Animal and Plant Health Inspection Service, Department of Agriculture, Ames, IA, United States; ^4^Subdirección General de Sanidad e Higiene Animal y Trazabilidad, Dirección General de Sanidad de la Producción Agraria, Ministerio de Agricultura, Pesca y Alimentación, Madrid, Spain

**Keywords:** *Mycobacterium bovis*, bovine tuberculosis, whole-genome sequencing, cattle-wildlife interface, surveillance, phylodynamics, interspecies transmission

## Abstract

Despite the efforts invested in the eradication of bovine tuberculosis in Spain, herd prevalence has remained constant in the country during the last 15 years (~1.5–1.9%) due to a combination of epidemiological factors impairing disease control, including between-species transmission. Here, our aim was to investigate the molecular diversity of *Mycobacterium bovis* isolates belonging to the highly prevalent SB0339 spoligotype in the cattle-wildlife interface in different regions of Spain using whole-genome sequencing (WGS). Genomic data of 136 *M. bovis* isolates recovered from different animal species (cattle, wild boar, fallow deer, and red deer) and locations between 2005 and 2018 were analyzed to investigate between- and within-species transmission, as well as within-herds. All sequenced isolates differed by 49–88 single nucleotide polymorphisms from their most recent common ancestor. Genetic heterogeneity was geographic rather than host species-specific, as isolates recovered from both cattle and wildlife from a given region were more closely related compared to isolates from the same species but geographically distant. In fact, a strong association between the geographic and the genetic distances separating pairs of *M. bovis* isolates was found, with a significantly stronger effect when cattle isolates were compared with wildlife or cattle-wildlife isolates in Spain. The same results were obtained in Madrid, the region with the largest number of sequenced isolates, but no differences depending on the host were observed. Within-herd genetic diversity was limited despite the considerable time elapsed between isolations. The detection of closely related strains in different hosts demonstrates the complex between-host transmission dynamics present in endemic areas in Spain. In conclusion, WGS results a valuable tool to track bTB infection at a high resolution and may contribute to achieve its eradication in Spain.

## Introduction

*Mycobacterium bovis*, a member of the *Mycobacterium tuberculosis* complex (MTBC) and the main causative agent of bovine tuberculosis (bTB), can infect a wide range of mammalian species, including domestic and wildlife species, as well as humans (Cousins, 2001; Aranaz et al., 2004; Perez-Lago et al., 2014). While the disease has been successfully eradicated in several countries using test and slaughter strategies and movement restrictions (Reviriego Gordejo and Vermeersch, 2006; EFSA Panel on Animal Health and Welfare (AHAW), 2014; More et al., 2015), bTB herd prevalence is still relatively high in several regions of the world (Guta et al., 2014a; Allen et al., 2018). Eradication efforts have been steadily hampered by the long latency period characteristic of the disease (Goodchild and Clifton-Hadley, 2001), the limitations of available diagnostic tests, particularly for the detection of early infection stages (de la Rua-Domenech et al., 2006), and the existence of wildlife reservoirs (Garnett et al., 2002; O’Brien et al., 2006). For this reason, in locations where wildlife reservoirs are known to be important in bTB transmission, disease control should be focused on an integrated approach at the cattle-wildlife interface.

In Spain, even though herd prevalence has decreased significantly since the initial implementation of the national eradication programs in the 80s, complete eradication of the disease remains elusive (2.2% herd prevalence in 2002 and 1.6% in 2020; Informe Final Técnico Tuberculosis Bovina Año, 2020). Bovine TB is not uniformly distributed across the country, as herd-prevalence is higher in central and southern regions (up to 10.9 and 6.4% in Castilla-La Mancha and Andalucia, respectively in 2020) while disease is virtually absent in north-western parts of Spain (0% herd-prevalence in Galicia, Asturias and the Canary Islands among others; Informe Final Técnico Tuberculosis Bovina Año, 2020). In bTB endemic areas the geographic distribution of livestock and wildlife species known to be infected with *M. bovis* often overlap. This is particularly relevant in the case of extensively managed livestock, which may share pastures and/or watering points with wildlife species, thus leading to a potential risk of interspecies transmission of the infection. Furthermore, in certain cases residual infection may persist over time in a herd in spite of the application of test-and-slaughter programs due to the presence of infected but undetected animals (Karolemeas et al., 2011; Guta et al., 2014b). Although wild boar is the most important wildlife reservoir of bTB in central and southern Spain, red deer and fallow deer may also serve as potential hosts (Gortazar et al., 2008; Naranjo et al., 2008).

Direct Variable Repeat spacer oligonucleotide typing (DVR spoligotyping) and Mycobacterial Interspersed Repetitive Unit - Variable Number of Tandem Repeat (MIRU-VNTR) typing are the most widely used techniques to characterize *M. bovis* isolates (Kamerbeek et al., 1997). Most *M. bovis* spoligotypes in Spain are circumscribed to specific regions, while a small proportion of highly prevalent profiles are widespread in the country (i.e., SB0121, SB0134 or SB0339; Rodriguez-Campos et al., 2012). Molecular typing of *M. bovis* strains has demonstrated that both cattle and wildlife species share similar spoligotype and VNTR profiles, with spatial clustering across hosts suggesting interspecific transmission at local scales (Aranaz et al., 2004). Under these circumstances, effective control of bTB can only be achieved if wildlife is also considered in eradication efforts. Furthermore, molecular characterization can help to determine if newly detected animals in previously infected herds may represent a reinfection or a failure removing all infected cattle in the previous herd-tests.

In spite of the widespread use of spoligotyping, this tool by itself is unable to precisely reconstruct the phylogeny of *M. bovis* isolates or differentiate epidemiologically unrelated isolates in certain settings given its limited resolution and high homoplasy rate (Comas et al., 2009). Recent advances in whole-genome sequencing (WGS) allow the performance of genome-wide screenings to study microbial populations, increasing the power of molecular epidemiology studies. In this sense, the use of single nucleotide polymorphisms (SNP)-based genotyping can provide valuable insights into the pathogenicity and evolution of *M. bovis* strains, allowing the identification of host or spatial associations, improve outbreak investigations and the differentiation of *M. bovis* strains into lineages and related phylogenetic structures (Joshi et al., 2012; Hauer et al., 2015; Ghebremariam et al., 2016; Trewby et al., 2016; Orloski et al., 2018; Price-Carter et al., 2018).

In the context of bTB eradication and control, tracking within-herd *M. bovis* variability is crucial as it may provide insights into possible new introductions in herd recurrence events. Additionally, understanding bTB transmission patterns within cattle and between cattle and wildlife species from different areas in Spain is crucial to assess the risk of infection to livestock at a multi-host interface, particularly in endemic areas where infected livestock and wildlife coexist. To date, no multi-region studies to characterize the genetic diversity among *M. bovis* isolates from cattle and wildlife in Spain using WGS have been performed. For these reasons, the aim of this study was to use WGS technologies to assess the risk of bTB transmission between cattle and wildlife using one of the most prevalent spoligotypes in Spain, SB0339, as a working example. This spoligotype is widely distributed across the country and is especially clustered in the province of Madrid, with >48% of the total SB0339 isolations (extracted from mycoDB.es). The specific objectives were (I) to investigate the genomic diversity among SB0339 *M. bovis* isolates recovered from cattle and wildlife during 2005–2018 from different regions of Spain in general, and in Madrid in particular; (II) to reconstruct the phylogenetic relationships between the isolates; and (III) to perform a comparative analysis of intraspecies and interspecies genomic diversity to understand the underlying evolutionary processes of *M. bovis* in the cattle-wildlife interface in Spain.

## Materials and Methods

### Isolate Selection and Laboratory Methods

As part of the bTB eradication program, tissue samples from cattle or wildlife suspected to be infected with bTB due to positive results in ante-mortem tests (cattle) or detection of granulomatous-appearing lesions in post-mortem inspection (cattle and wildlife) are collected and submitted to regional Reference Laboratories for bacteriological culture. Once bacterial growth is detected, DNA is extracted from cultures and the presence of MTBC members is confirmed through conventional molecular methods, such as PCR or DVR spoligotyping (Cousins et al., 1991; Kamerbeek et al., 1997). In this context, a selection of *M. bovis* isolates recovered from naturally infected cattle and wildlife tissue samples using standard isolation procedures at the VISAVET Health Surveillance Center were considered for inclusion in the analyses performed here.

The SB0339 was selected for the study as this pattern is the third most prevalent spoligotype in Spain [8.1% of all isolates, only preceded by SB0121 (28.2%) and SB0134 (10.7%)], and the most frequently isolated spoligotype in Madrid (Rodriguez-Campos et al., 2010, 2012). Moreover, this spoligotype is frequently detected in both cattle and wildlife (i.e., red deer, fallow deer and wild boar). A total of 1,501 (44% out of the total SB0339 strains in Spain, mycoDB.es) SB0339 samples were available for selection at the VISAVET Health Surveillance Centre strain collection and were divided into strata based on the animal species of isolation, location (province), and year of isolation. Subsequently, a stratified random sampling was performed, so that only subsets of SB0339 isolates recovered from provinces and years in which more than one host species was available were further considered for selection. Accordingly, 259 isolates of the SB0339 spoligotype recovered from cattle and wildlife between 2005 and 2018 in 21 regions in Spain were subjected to bacteriological re-culture. Additionally, to evaluate the degree of genetic heterogeneity among *M. bovis* isolates in bTB infected herds, between 2 and 8 isolates from 15 herds were selected. Ten of these herds had been chronically infected, defined as herds with *M. bovis* isolations in more than 1 year.

Selected isolates identified as *M. bovis* were centrifuged at 2,500 × *g* for 10 min and subsequently washed twice with 5 ml of phosphate-buffered saline (PBS, Gibco) and centrifuged. Supernatants were poured off and the pellets were re-suspended in 4 ml of PBS. Samples were inactivated and mycobacterial DNA was further separated from the other cellular components using a bead disruption and phenol/chloroform/isoamyl alcohol (PCI, Sigma-Aldrich) based protocol as described elsewhere.^1^ The quality and concentration of DNA was measured using a nanodrop spectrophotometer and a Qubit^™^ fluorometer (Invitrogen).

### Whole-Genome Sequencing

The extracted mycobacterial DNA was submitted to the National Veterinary Services Laboratory (NVSL) in Ames, Iowa (United States) to perform WGS. Libraries were prepared using Nextera XT preparation kit and the total genomic DNA was sequenced on a MiSeq instrument to produce 2 × 250 bp reads (Illumina, San Diego, CA, United States). Generated FASTQ files were analyzed using the United States Department of Agriculture (USDA) NVSL in-house vSNP pipeline, a high-resolution reference dependent variant calling pipeline.^2^ Briefly, genomic reads were mapped against the reference genome *M. bovis* AF2122/97 (National Center for Biotechnology Information [NCBI] accession number NC_0002945) using the Burrows-Wheeler Aligner (BWA; Li and Durbin, 2009; Malone et al., 2017), and vSNP then called SNPs relative to the reference. Defining SNPs were used to identify different groups of isolates within the vSNP pipeline as specified in vSNP dependency files.

Single nucleotide polymorphisms were called using FreeBayes, a haplotype-based variant detector, generating variant call format files (VCF; Garrison and Marth, 2012). Results were filtered using a minimum Phred-scaled quality (QUAL) score of 150 and an Allele Count (AC) of 2. Isolates that contained heterologous/heterozygous calls at a SNP position (AC = 1 and present in <90% of the reads) were considered ambiguous as coded by the International Union of Pure and Applied Chemistry (IUAPC) and were visually inspected. Those SNP positions that had a variant call in more than 90% of reads were considered homozygous whereas variant calls identified in <90% of the reads the sample were considered heterozygous and removed from the analysis.

A summary of quality metrics was then generated to evaluate the performance of the sequencing run of each isolate. This included the average depth of coverage, the average read length, the percent of the reference genome covered by the reads from each isolate, the number of contigs not mapping to the reference, the number of SNPs with a quality (QUAL) score of >300 with an AC of 2 (good SNPs), and the spoligotype octal code. The octal code was based on the counts of each spacer sequence against the raw FASTQ files. Reads identified as *M. tuberculosis* complex were isolated and cleaned reads were ran through vSNP.

The SNPs tables and the phylogenetic trees were created after removing all uninformative SNPs (i.e., homogeneous/monomorphic between the isolates). Those SNPs identified in the ∼10% of the genome composed of repetitive regions were excluded using default masking files in the vSNP dependencies, as mapping in these regions is error prone (Cole et al., 1998). This included the highly GC-rich and polymorphic proline-glutamate (PE)/proline-proline-glutamate (PPE) gene family (Cole et al., 1998). Additionally, SNPs present in areas with an anomalous accumulation of variants (typically ≥2 SNPs in 10 bp due to poor alignment) were omitted. After removing all uninformative and potentially erroneous variant positions, informative SNPs were validated by visualizing alignment files in the Integrative Genomics Viewer (IGV) software (Robinson et al., 2011).

Maximum likelihood phylogenetic trees were built with RAxML (Stamatakis, 2014) using the SNP alignment file containing the concatenated polymorphic and validated SNPs. A general time reversible (GTR) CAT model for the nucleotide substitution rate with a Gamma distribution was assumed to account for between-site heterogeneity (Stamatakis, 2014). The accuracy of the phylogenetic tree was confirmed using the manually validated SNP table, and additional filtering of questionable SNPs was performed on an isolate-by-isolate basis when appropriate.

The degree of genetic relatedness among *M. bovis* genomes was assessed between- and within-species using the pairwise genetic distances between isolates. Isolates were identified using the year of isolation followed by the isolate number (#), the initial(s) of the province of origin, the animal species, and, if applicable, the code of the herd or the estate from which the sample was recovered.

### Between- and Within-Species Phylogenetic Analyses

The phylogenetic analysis was first performed on the total number of isolates available for the study. The distributions of the pairwise SNP distances between and within cattle and wildlife isolates were compared using the Kruskal-Wallis test followed by post-hoc tests with Bonferroni corrections for multiple comparisons. Hierarchical Bayesian clustering was performed to determine the population structure using BAPS (Cheng et al., 2013). Isolates were considered genetically close when their sequences were within 0–3 SNPs from each other (Hatherell et al., 2016). The geographic distance in kilometers between pairs of isolates was computed using the centroids of the municipalities in which the isolates were recovered, and the relationship between genetic and geographic distances was assessed using Spearman’s rho correlation test.

Subsequently, and to avoid an artificial increase of cattle within-species homogeneity, only one isolate per herd was randomly selected. The relationship between the genetic distance between two isolates and the distance between their geographic origins (province, municipality and/or state) was then explored using linear regression models that also considered the host species of origin (i.e., if the pair of isolates both originated from cattle, from wildlife or from both). The model considered the selected risk factors (geographic distance in km and host species) along with the interaction between the two. Finally, the same analysis was performed only on isolates from the Madrid region, from which the largest number of isolates was available (see results). Since only one isolate per herd was randomly selected in cattle herds with multiple isolates, the impact of the selection on the assessment of the association between the genetic distance and the geographical origin and host species was evaluated through a sensitivity analysis consisting in repeating 10 times the analyses with one isolate per intensively sampled herd selected at random.

### Within- and Between-Herd Phylogenetic Analyses

Isolates recovered from different cattle herds were selected to evaluate the genetic variability of isolates retrieved from the same herds over time. Data on the time elapsed between the isolates were recovered and the number of years with isolations for each chronically infected herd was available. Within-herd genetic distances (i.e., between isolates originating from a single herd) considering all herds and only those chronically infected (with bTB isolations retrieved in more than 1 year) were assessed, along with the number of closely related (≤3 SNPs) strains present in the herds for different time periods. The correlation between median genetic distances and the number of different years from which isolates were retrieved in each herd was calculated.

Multiple comparisons, graphics and phylogeny analyses were conducted using the ggplot2 (Wickham, 2009), dplyr (Wickham et al., 2019), ape (Paradis et al., 2004), rgdal (Bivand et al., 2013), rhierbaps (Tonkin-Hill et al., 2018), and PMCMRplus (Pohlert, 2020) packages for R software (R Core Team, 2019).

## Results

### Descriptive Analysis

**Figure 1:**
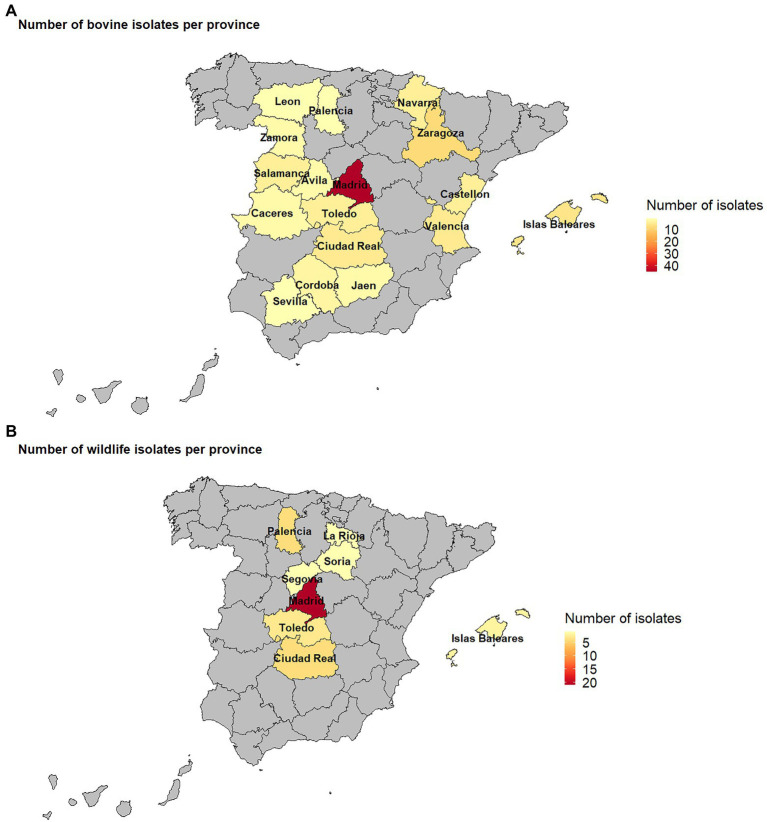
Map of the number of *M. bovis* SB0339 isolates recovered from cattle **(A)** and wildlife **(B)** per province included in the study.

One hundred and thirty-six isolates were recovered out of the 259 SB0339 selected samples. Out of these, 72.8% (*n* = 99) derived from the VISAVET strain collection, while the remaining 27.2% were provided by other Regional Laboratories. These 136 strains were obtained during the 2005–2018 period from cattle (72.8%, *n* = 99), wild boar (14.7%, *n* = 20), fallow deer (6.6%, *n* = 9), and red deer (5.9%, *n* = 8, Table 1). A median number of 10 samples per year (range 2–25) were available, with more isolates included after 2009, and years 2010 and 2017 accounting for the highest number of isolates (*n* = 17 and *n* = 25, respectively, Supplementary Figure S1). The areas from which the samples used in the study originated included the northwestern, northeastern, central, eastern, and southern regions of the country (Table 1; Figure 1). Individual isolate information and associated metadata are detailed in Supplementary Table S1. The highest (48.5%, *n* = 66) proportion of isolates was derived from bTB infected animals located in Madrid, followed by Ciudad Real (6.6%, *n* = 9), Islas Baleares (from now on, the region of Mallorca, 5.9%, *n* = 8), Zaragoza (5.9%, *n* = 8) and Toledo (5.1%, *n* = 7). The rest of the provinces accounted for the remaining 38 (27.9%) isolates (Table 1; Figure 1). Information on the exact municipality from which isolates originated was available in 121 isolates (89%).

**Table 1:** Number of cattle, red deer, wild boar, and fallow deer sequenced isolates per province and overall included in the study.

		Number of Isolates					
	Province/ region code	Total	%	Cattle	Red deer	Wild boar	Fallow deer
Avila	A	2	1.5	2			
Caceres	CC	2	1.5	2			
Castellon	CS	4	2.9	4			
Ciudad Real	CR	9	6.6	5	4		
Cordoba	CO	3	2.2	3			
Jaen	J	2	1.5	2			
La Rioja	LR	1	0.7			1	
Leon	LE	1	0.7	1			
Madrid	M	66	48.5	45	2	12	7
Mallorca	MA	8	5.9	6			2
Navarra	NA	4	2.9	4			
Palencia	PA	5	3.7	1		4	
Salamanca	SA	4	2.9	4			
Segovia	SG	1	0.7		1		
Sevilla	SE	1	0.7	1			
Soria	SO	1	0.7			1	
Toledo	TO	7	5.2	4	1	2	
Valencia	V	5	3.7	5			
Zamora	ZA	2	1.5	2			
Zaragoza	Z	8	5.9	8			
TOTAL		136	100	99	8	20	9

The largest number of both cattle (45.5%, *n* = 45/99) and wildlife (56.8%, *n* = 21/37) isolates were collected from herds and estates located in Madrid, where wild boar was the main wildlife species sampled (*n* = 12/21, Table 1; Figure 1). Out of the 7 municipalities in Madrid from which isolates were included in the study (Colmenar Viejo, Madrid, El Boalo, Quijorna, Rascafria, San Agustin de Guadalix, and San Sebastian de los Reyes), Colmenar Viejo and Madrid accounted for the highest number of cattle (*n* = 26 and *n* = 13, respectively) and wildlife (*n* = 8 in each) isolates (Supplementary Table S1).

### *Mycobacterium bovis* Genomic Data

Average depth of coverage ranged between 20x and 174x except 7 isolates, with 85.3% (*n* = 116) of the isolates with values ≥30x. The average read length of the isolates ranged between 186.5 and 239.4 (median = 229.7, IQR = 222.6–233.6). The median genome coverage was 99.0% (IQR = 98.9–99.1). Alignment summary data and sample associated metadata of the 136 isolates subjected to WGS are included in Supplementary Table S1. No indication of the presence of mixed isolates was detected in the sequenced population as not many occurrences of mixed positions were observed recurrently across the genome in regions with good mapping scores.

### Phylogenetic Analysis and Clustering Structure

**Figure 2:**
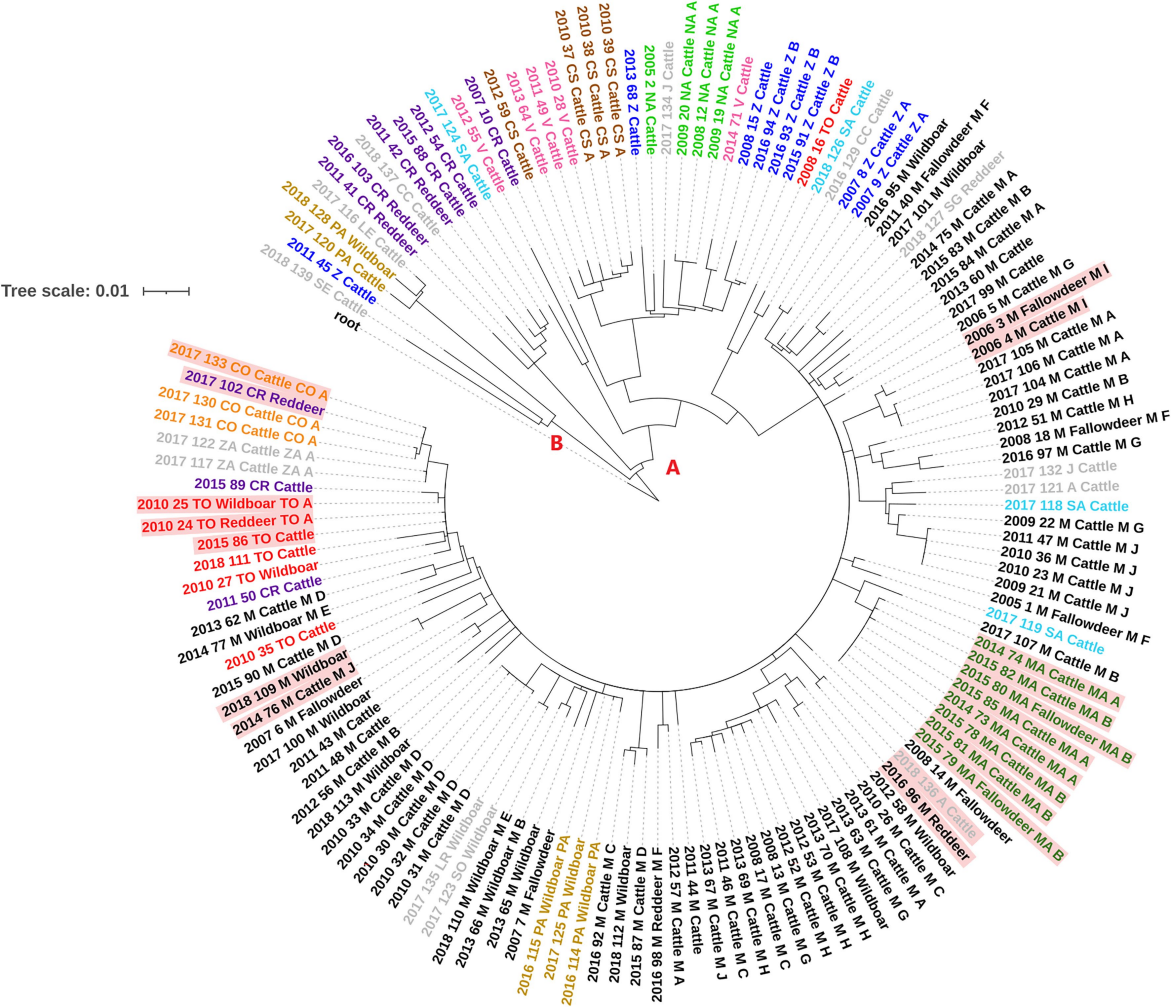
Whole-genome sequence RAxML phylogenetic tree constructed using a GTR-CAT model of 136 Spanish *M. bovis* SB0339 samples. The two distantly related groups of isolates are indicated with letters A and B. Isolate tags are colored based on the province of isolation which codes are shown in Table 1. Isolates with genetic distances of ≤3 SNPs between cattle and wildlife are colored in red background.

One-hundred and eleven SNPs were excluded as these were detected in regions where mapping was error prone or were present in areas with an anomalous accumulation of variants. The analysis of the informative SNPs showed that among the studied Spanish *M. bovis* isolates there were a total of 1,345 SNPs, of which 773 were singletons (present in a single isolate). Genetic diversity among isolates was variable, with an average number of 62 SNPs (range = 49–88) since diverging from their most recent common ancestor (MRCA). Isolates were subdivided into two distantly related groups (A and B, Figure 2), with the largest one (A) further subdivided into six different clades (A.1-A.3, A.4.1, A.4.2 and A.5) according to BAPS and based on a maximum within-clade distance of 57 SNPs (Supplementary Table S2; Figure 2; Supplementary Figures S2, S3). All isolates in group A were characterized by the presence of two additional SNPs (absent in group B) and included 96% (*n* = 95) of the cattle and 97.3% (*n* = 36) wildlife isolates. Group B included 5 isolates of which one was recovered from a wild boar and the remaining from cattle.

Distribution of clades per province is shown in Supplementary Figure S4. Clade A.2 represented the largest clade/number of isolates (74.3% of the total) and included all bovine and wildlife isolates from Madrid (*n* = 66) and Mallorca (*n* = 8), together with 27 bovine and wildlife isolates from 12 different provinces (Supplementary Table S2; Supplementary Figure S4). Isolates in this clade were recovered throughout the whole study period (2005–2018) with a median number of seven isolates per year (range 1–21). Clade A.1 included 3 bovine and 3 wildlife isolates recovered between 2011 and 2018 from provinces located in central and southern Spain, whereas isolates in clades A.3, A.4.1, A.4.2 and A.5 were exclusively from cattle and were cultured in 2007, 2008, 2016 and 2018 (clade A.3, *n* = 4), 2010–2012 (clade A.4.1, *n* = 7), 2005–2017 (clade A.4.2, *n* = 11) and 2012 and 2017 (clade A.5, *n* = 2). Isolates from clades A.3, A.4.1 and A.4.2 were recovered from provinces that covered the central strip from West to East of Spain and two northern regions, while the two isolates in clade A.5 originated from very distant (>600 km) provinces (Supplementary Figure S4). Finally, isolates in group B were recovered in 2011 (*n* = 1) and 2017–2018 (*n* = 4).

Between-group (A versus B) genetic distance ranged between 115 and 163 SNPs, whereas within-group genetic distances were much lower in group A (median = 37 SNPs, range = 0–129) than in group B (median = 96 SNPs, range = 12–108, Supplementary Table S2). Median within-clade genetic distance in group A was 30 (range = 21–57; Supplementary Table S2), whereas between-clades median genetic distance was 85 (range = 35–114). Although isolates in group B were genetically distant (median distance 96 SNPs), two cattle and one wild boar isolates were relatively closely related (<16 SNPs) and recovered close in time (2017 and 2018).

### Between- and Within-Species Genetic Diversity

Genetic diversity between isolates from different hosts was assessed using 90 isolates (53 from cattle -including only one per herd- and all wildlife isolates: 20 from wild boar, 9 from fallow deer, and 8 from red deer).

Six events of short (≤3 SNPs) genetic distances between isolates from different host species recovered close in time (within 5 years, and within <2 years in five of them) and space (within the same province or neighboring provinces) were found. Specifically, these events included: one cattle and one red deer isolates recovered in Avila and Madrid, respectively, one cattle isolate from Cordoba and a red deer sample from Ciudad Real, a triplet formed by one cattle, a wild boar and one red deer *M. bovis* isolates from Toledo, two *M. bovis* samples isolated from cattle and fallow deer recovered in the same municipality in Madrid, a pair of cattle and wild boar isolates retrieved from two distantly located regions of Madrid, and a cluster of cattle from two chronically infected herds (herds MAA and MAB) and 2 fallow deer isolates recovered in Mallorca (Figure 2).

**Figure 3:**
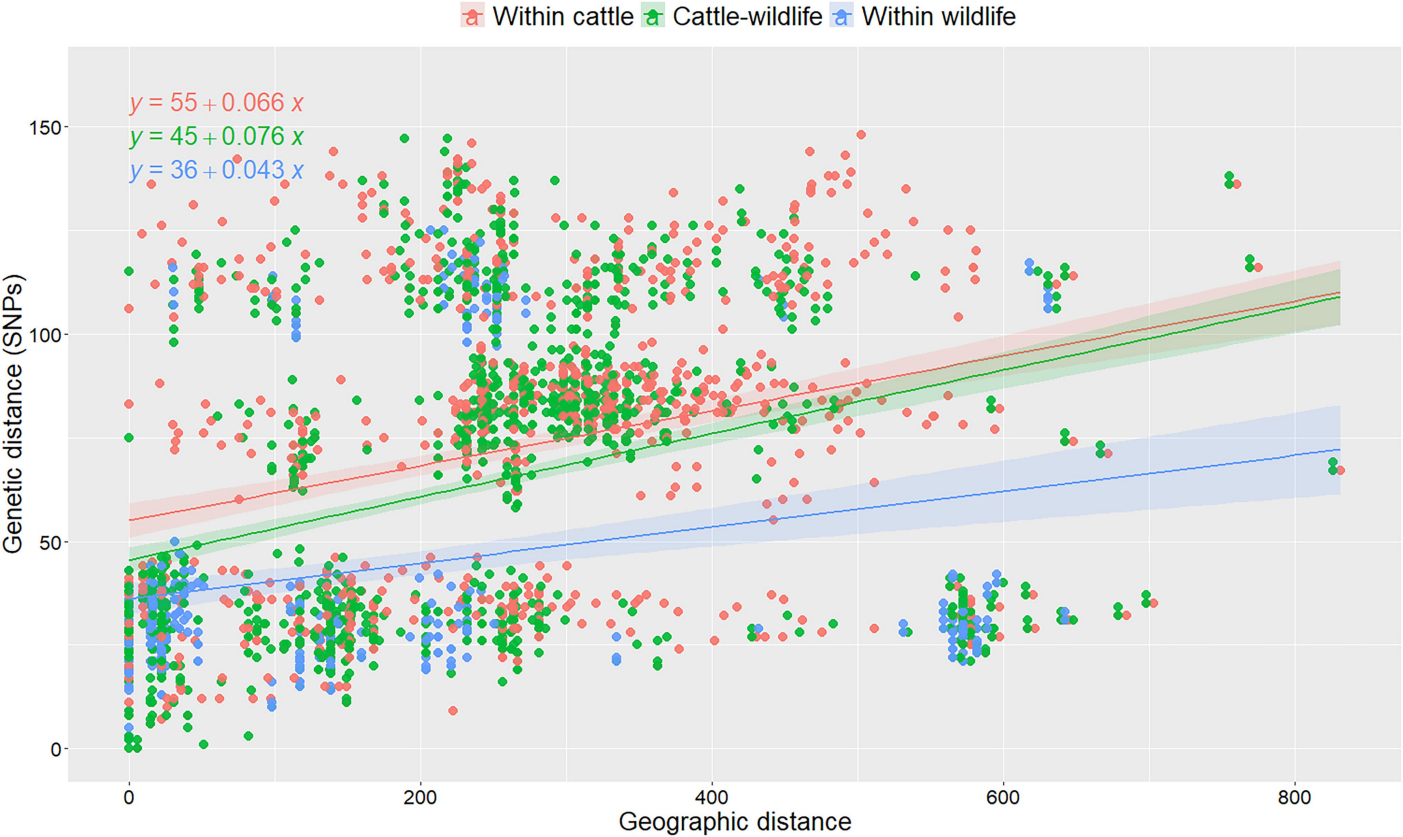
Analysis of pairwise genetic distances between isolates recovered within- and between-species as a function of their geographic distances of isolation in one out of the 10 replicas based on random selection of one isolate per chronically infected herd. Linear regression lines were fitted to denote the relationship between genetic and geographic distances per animal species combination and formulas were depicted for each category. Colors denote different combinations of pairs of isolates retrieved from cattle (red), wildlife (green) and collected from cattle and wildlife (blue). The mathematical equations were depicted for each animal species combination.

Overall, median genetic divergence within cattle isolates in Spain (median = 80 SNPs, IQR = 36–109), was significantly (*p* < 0.001, Kruskal-Wallis) higher than between cattle and wildlife (median = 66 SNPs, IQR = 29–93), and within-wildlife isolates (median = 29 SNPs, IQR = 22–40). No changes were observed when the cattle within-species variability was reassessed using 10 random selections of isolates from the herds with multiple isolates available (maximum percentage change 0.01%). Genetic distances were low-to-moderately correlated with geographic distances (rho = 0.42, *p* < 0.001). When the association between genetic and geographic distances was analyzed considering the host from which isolates originated using a linear model, both the host and the interaction between host and geographic distance was significant (Supplementary Table S3): genetic distance increased with increasing geographic distances between the location from which the two isolates originated, but this effect was less pronounced when considering two cattle isolates compared to when two wildlife isolates were selected (and similar to when considering pairs retrieved from both cattle and wildlife; Figure 3). A 5 SNP increase in the genetic diversity within-wildlife isolates was expected for every 100 km increase between the origin of each pair of isolates, whereas this number increased to 7 when both isolates were recovered from cattle (Supplementary Table S3; Figure 3). Coefficients and slopes from the 10 models built based on the different random selections of cattle isolates from chronically infected herds were similar (<10% change), suggesting that the random selection of isolates in intensively sampled herd had no substantial impact on the observed genetic diversity (Figure 3; Supplementary Figure S5; Supplementary Table S3).

In contrast, when the same analysis was performed in the subset of isolates recovered in Madrid, no significant (*p* > 0.05, Kruskal-Wallis test) differences in the distances found between isolates from the same species (median range within-cattle = 30–32 SNPs; median within-wildlife = 30 SNPs) or different species (median range cattle-wildlife = 29–31 SNPs) were identified. The final model included only the geographic distance between isolates as a covariable as no significant effect of the animal species was found. An increase of 13 SNPs per 100 km distance between pairs of isolates was observed based on the results of the 10 linear regression models (Supplementary Table S4). Coefficients of the regression models were more susceptible to variation based on the randomly selected isolates per herd (up to 20% change, Supplementary Table S4).

### Within- and Between-Herd Genetic Diversity

Among the 99 cattle sequences, 61 originated from the same 15 herds and were selected to evaluate within-herd diversity (Table 2). Median number of isolates originating from the 15 herds was 3 (IQR = 3–5) and were retrieved in a median of 2 different years (IQR = 1–4). Ten of these herds were considered as chronically infected (isolates retrieved in >1 year), yielding 48 cattle isolates (median number of isolates per chronically infected herd = 5, range = 3–8) recovered throughout a median of 4 different years (IQR = 2–5). The remaining (*n* = 5) non-chronically infected 5 herds accounted for 2–3 samples recovered in one single year (Table 2).

**Table 2:** Herd identification, province of origin, number of isolates, years between first and last isolations, median number of isolates recovered per year, and % of isolates within a ≤ 3-SNPs genetic distance in herds included in the analysis of within-herd *Mycobacterium bovis* genetic diversity.

Herd ID	Group/Clade	Province	Chronic herd	Number of years with *M. bovis* isolates	Number of isolates	First and last year of isolation	Median number of isolates/year	Median genetic distance (IQR)	% of isolates within a ≤ 3-SNPs genetic distance
M_A	A.2	Madrid	YES	5	7	2012/2017	1	34 (31–37)	42.9
M_J	A.2	Madrid	YES	5	6	2009/2014	1	44 (1–46)	66.7
M_G	A.2	Madrid	YES	5	5	2006/2016	1	32 (23–38)	0.0
M_B	A.2	Madrid	YES	4	4	2010/2017	1	32 (30–33)	0.0
M_C	A.2	Madrid	YES	4	4	2008/2016	1	29 (20–37)	0.0
M_D	A.2	Madrid	YES	3	8	2010/2015	2	30 (1–34)	62.5
M_H	A.2	Madrid	YES	2	5	2012/2013	3	9 (6–29)	0.0
MA_A	A.2	Mallorca	YES	2	3	2014/2015	2	2 (1–2)	100.0
NA_A	A.4.2	Navarra	YES	2	3	2008/2009	2	2 (1–2)	100.0
Z_B	A.4.2	Zaragoza	YES	2	3	2015/2016	2	13 (7–13)	66.7
CO_A	A.2	Cordoba	No	1	3	2017	–	2 (2–3)	100.0
MA_B	A.2	Mallorca	No	1	3	2015	–	1 (1–1)	100.0
CS_A	A.4.2	Castellon	No	1	3	2010	–	6 (6–7)	0.0
Z_A	A.2	Zaragoza	No	1	2	2007	–	3 (3–3)	100.0
ZA_A	A.2	Zamora	No	1	2	2017	–	0	100.0
Total					61				

Isolates from the within-herd genetic analyses were all included in three clades from group A (A.2, A.4.1 and A.4.2). Median within-herd genetic distances among the isolates from each of the 15 herds ranged between 0 and 44 SNPs (median = 9). A higher genetic diversity was observed in isolates recovered from herds located in Madrid (namely, M_A-M_C, M_G, and M_H), although isolates from single herds tended to cluster together (Supplementary Figure S6). Isolates recovered in herd CS_A, recovered within the same year, were in a range of 5–7 SNPs apart. The genetic distance between isolates recovered from the 10 chronically infected herds was 2–44 SNPs (median = 30), whereas genetic diversity was lower in the isolates from the remaining 5 herds that were not chronically bTB infected (median within-herd genetic distance = 2 SNPs, range = 0–6).

**Figure 4:**
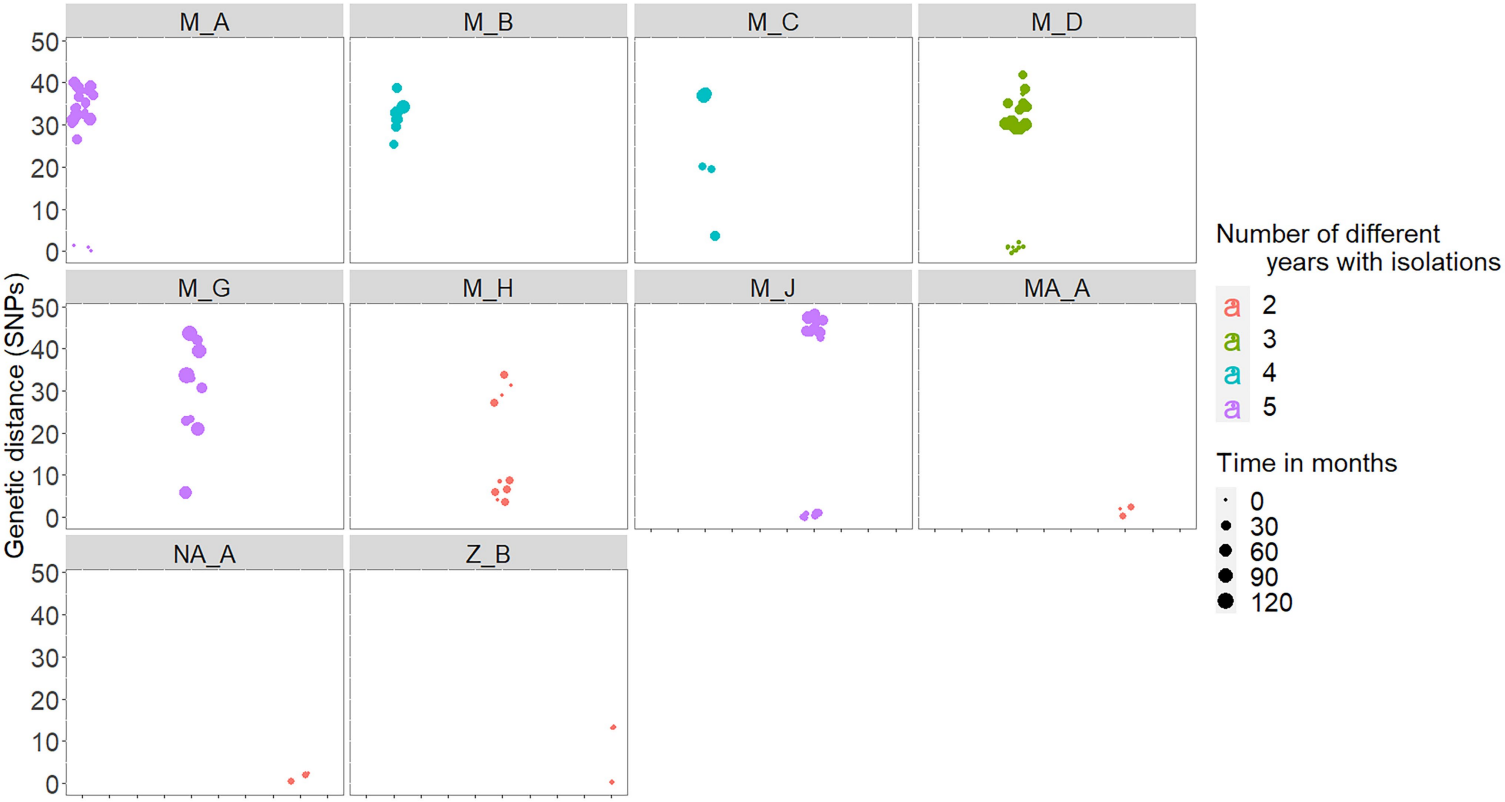
Within-herd pairwise genetic distances among isolates recovered in the 10 chronically infected herds. Genetic distances are colored based on the number of different years with *M. bovis* isolates (between 2 and 5 different years), and the size represents the number of months elapsed between each pair of isolates.

In five out the 10 chronically infected herds, at least 60% of the isolates recovered in a range of 2–5 different years were within 3 SNPs distance with each other (Table 2). In fact, there was a high positive correlation between median genetic distances and number of different years from which isolates originated in a herd (rho = 0.88, *p* < 0.001). Six (M_A, M_C, M_D, M_H, and M_J) out the seven chronically infected herds located in Madrid included similar (i.e., ≤3 SNPs), nearly similar (i.e., 4 SNPs for M_C and M_H) and different (i.e., >20 SNPs) isolates recovered close (same year) and distant (up to 10 years apart) in time, respectively (Figure 4; Table 2). Nevertheless, two of these herds (M_D and M_H) yielded isolates separated by genetic distances that ranged between 29 and 37 SNPs that were recovered within the same month (Figure 4). This pattern of bimodal distribution of within-herd pairwise genetic distances was not evident in the remaining chronic herd from Madrid and chronic herds located in other regions (namely, M_B, MA_A, NA_A, and Z_B, Figure 4). Additionally, an event of potential persistence or re-introduction of similar strains was identified in herd M_G, as shown by closely related (6 SNPs) strains in the herd found 5 years apart (Figure 4).

## Discussion

In this study, WGS information from a large panel of *M. bovis* isolates recovered from several animal species and regions in Spain was used to understand the potential for between- and within-species transmission and to elucidate the role of wildlife in bTB transmission. This is the first large scale genomic study describing *M. bovis* diversity at the livestock-wildlife interface from multiple provinces/regions in Spain and highlights the relevant role that genomics and phylogenetic approaches can have to gain knowledge on bTB epidemiology.

We selected SB0339 isolates as this was the third most frequent spoligotype in Spain, and the most frequently retrieved in both cattle and wildlife populations. It has been isolated recurrently mainly from the south-central and northern regions of the country, with evidence of interspecies transmission (Garcia-Jimenez et al., 2013). Although other spoligotypes have been also found repeatedly in the cattle-wildlife interface (e.g., SB0121 and SB0134), more than one third of all SB0339 isolates originate from wild boar, red deer, and fallow deer (largest proportion of wildlife isolates for a given spoligotype in Spain; mycoDB.es). Additionally, SB0339 represents the most frequently isolated molecular pattern in Madrid, including >50% of all *M. bovis* samples recovered in the region (mycoDB.es).

The highest (72.8%) proportion of isolates included in this study were recovered from cattle, whereas 37 SB0339 isolates were recovered from different wildlife species. More than half of them (*n* = 20) were recovered from wild boar, which is considered the main maintenance host in the Mediterranean Iberia (Munoz-Mendoza et al., 2013; Gortazar et al., 2015). Isolates included in the study were sampled at different points in time (heterochronous data) between 2005 and 2018. This wide time interval was selected given the small mutation rate of *M. bovis* [median estimates between 0.15 and 0.5 events per genome per year (Biek et al., 2012; Trewby et al., 2016; Crispell et al., 2017)], which makes necessary to compare isolates recovered through long periods when attempting to reconstruct the phylogenetic relationships between them. Out of the total sequenced samples, 84.6% (*n* = 115) were recovered after 2009, as viable and high-quality DNA was difficult to retrieve from samples (especially wildlife) recovered during the 2005–2009 period. No isolates prior to 2005 were available at the VISAVET Centre.

Genome-wide data of the 136 bTB isolates revealed a large amount of acquired SNPs within groups A and B, with isolates being 47–72 and 38–61 SNPs apart from their MRCA, respectively (data not shown). Additionally, seven distinct clades could be distinguished among the sequenced collection, of which three contained both wildlife and cattle-derived *M. bovis* sequences. These results may not only be suggestive of a long history of endemicity, but also that *M. bovis* infection has been transmitted between cattle and wildlife populations in Spain for a long time. In fact, a high degree of genetic diversity was expected in SB0339 isolates, given that it represents 8% of all *M. bovis* isolates typed in Spain, and has been circulating in several areas of Spain over extended periods of time (Rodriguez-Campos et al., 2010; Gortazar and Boadella, 2014).

Pairwise genetic distances among isolates in the major clade A.2 were the lowest of all clades even though it included the highest number of sequences (74.3% of all the collection) and included isolates from both cattle and wildlife retrieved over a long time period. The similarity between strains in this clade recovered from different host species is suggestive of recent transmission events and/or transmission from a (non-sampled) common source. Evidence of *M. bovis* transmission between livestock and wildlife has been reported in numerous studies worldwide, especially in areas where several susceptible species coexist. A study including a total of 27 different spoligotypes conducted in Catalonia, a region located in Northeastern Spain, revealed transmission among different hosts (Perea et al., 2021). Research conducted in United Kingdom showed that badger isolates were in a range of 0–4 SNPs apart from the nearest cattle isolate, and that transmission occurred more frequently from badgers to cattle than vice versa (Biek et al., 2012; Crispell et al., 2019). Using a Bayesian approach, a recent study performed in France revealed a high rate of interspecies transmission between cattle and badger populations (Duault et al., 2022). Additionally, a high rate of genetic exchange between sampled livestock and wildlife populations was also suggested in New Zealand (Crispell et al., 2017).

Overall, time and geographical origin of isolates were good predictors of genetic distances regardless the host species of origin of the isolate. Genetic heterogeneity was geographic rather than host species-specific, as isolates recovered from different animal species within the same provinces tended to be more closely related than those originating from the same species and different provinces. However, despite the potential for interspecies transmission suggested by the findings in our study, the overall clustering of isolates by host species pointed that *M. bovis* strains recovered from wild hosts were less diverse than those coming from cattle, and that genetic distances were associated with the province of origin (Figure 2; Supplementary Table S2). This was particularly evident in the case of the wild boar samples, with isolates recovered either from the same or neighboring provinces being few SNPs away from each other. In fact, previous study conducted in Catalonia suggested that proximity and neighborhood were the two most important risk factors associated with the observed genetic heterogeneity (Perea et al., 2021). In our study, a substantially higher heterogeneity in the pairwise genetic distances among cattle isolates in Spain compared with isolates from wildlife and pairs from cattle and wildlife was observed, what could be due to the transmission of a larger pool of SB0339 strains in the cattle reservoir that do not reach wildlife (Figure 3; Supplementary Table S3). In contrast, in the analysis of strains from Madrid the genetic diversity of isolates recovered from cattle and from wildlife was more similar. Although a substantial genetic homogeneity at the spoligo-VNTR level was identified among cattle isolates in Madrid in a previous study (de la Cruz et al., 2014), genome-wide analyses performed here revealed an overall high degree of genetic heterogeneity irrespective of the host species of origin suggesting a long history of SB0339 endemic circulation from multiple sources in Madrid (Supplementary Table S4). However, no information on the VNTR profiles were available for this study, as VNTR typing is no longer routinely performed in our laboratory. Even though significant relationship between *M. bovis* genetic and geographic distance was identified in the region, the lack of association of the host species with the genetic distance separating two isolates recovered from Madrid may be also suggestive of evolution of strains in unsampled hosts not considered here (that could include cattle populations with no transmission linkage with wildlife, Supplementary Table S4; Price-Carter et al., 2018). In contrast, if only provinces with a large proportion of cattle and wildlife isolates (i.e., Madrid, Ciudad Real, and Toledo) were considered in the analysis, results obtained were the same as those of the overall analysis of Spain (data not shown). This analysis confirmed that, even after performing a more similar comparison of the genetic diversity in regions with a higher number of wildlife isolations, Madrid is representative of a convoluted and interspecific SB0339 transmission scenario. Additionally, the overall genetic diversity of isolates included in clade A.2 (Supplementary Figures S2, S4) revealed similar results as in Madrid, as no significant differences in the distances found between isolates from the same species (median range within-cattle = 31–32 SNPs; median within-wildlife = 27 SNPs) or different species (median range cattle-wildlife = 28–29 SNPs) were identified (data not shown). The large heterogeneity (range 55–163 SNPs) identified when clade A.2 was compared with the remaining clades identified in regions other than Madrid (Supplementary Table S2) concurs with the hypothesis of long history of endemicity of this spoligotype established in this study. Nevertheless, phylogenetic relationships described in this study may be particularly sensitive to selection bias in the sampled population of Madrid Vs. the overall sequenced isolates recovered in multiples provinces of Spain, and differences in the observed effect explained here should be interpreted with caution. In spite of the observed association between genetic and geographic distances, in several instances genetically unrelated (i.e., highly distinct) isolates were retrieved from close locations (and in some instances even from the same herd), suggesting that infection could have occurred in a different location, particularly in cattle, since most (>81%) wildlife isolates were retrieved from animals in fenced hunting states and thus limited animal movement was expected. Unfortunately, the individual movements of all sampled cattle were not available for this study, and therefore the hypothesis of new introductions due to movement of infected animals could not be confirmed.

Moreover, three out of the six groups of highly similar (≤3 SNPs) strains recovered from livestock and wildlife were found in Madrid (Figure 2; Supplementary Figure S2). In fact, the SB0339 pattern has been recurrently found in certain areas, especially in Monte de El Pardo Nature Reserve located in Madrid municipality and in Colmenar Viejo, where risk factors such as extensively managed herds and abundant presence of wildlife reservoirs may explain the observed interspecies genetic similarity and that bTB prevalence has remained historically high in both livestock and wildlife (Aranaz et al., 2004; Rodriguez-Campos et al., 2010).

The analyses of the within-herd genetic diversity in isolates from chronically infected herds were aimed at providing some information on the degree of difference expected over time in a given herd, what could help to differentiate the source of an outbreak (i.e., relapse Vs. reinfection; Hatherell et al., 2016). A ≤ 3 SNPs threshold was used here to define transmission clusters in which we are confident that similar strains are circulating in different hosts. This value was lower than that selected in the study performed in Catalonia, where a pairwise distance of ≤12 SNPs was considered to define putative transmission clusters based on the observed clustering patterns (Perea et al., 2021). In our study, isolates with ≤3 SNPs were considered closely related because most epidemiologically linked isolates recovered from the same herd and sampled within the same year did not exceed this level of divergence. When a ≤ 10-SNPs threshold was applied in our analyses, 2 additional herds (namely, CS_A and M_H) out of the 15 analyzed herds had >50% of the sampled strains within this cut-off circulating during the sampled period (Figure 4). The use of different thresholds may impact the inferences suggested here (i.e., relapse Vs. new introductions). However, our aim here was to identify clusters of molecular patterns that are likely to have arisen out of epidemiological links instead of the acquisition of SNPs derived from evolution process. Additionally, we selected this harsh cut-off as it concurred with that derived from extended literature review of the MTBC to accurately identify events of recent transmission, relapse, and re-infections (Kato-Maeda et al., 2013; Roetzer et al., 2013; Lee et al., 2015). Overall, we identified a strong relationship of time and spatial origin and genetic variability, so that those factors were highly informative to characterize circulation as isolates tended to cluster in a within-herd pattern. Genetic distances observed both between- and within-herds were highly heterogeneous depending on the epidemiological history (number of years with bTB isolations in the herd) as median pairwise genetic distances observed in the 10 chronically sampled herds varied largely (range 2–44 SNPs) compared with those that were not chronically bTB infected (range 0–6 SNPs). This was expected as non-chronically infected herds were sampled over a 1-year period Vs. a median number of 3.5 different years with isolations in chronically infected herds. However, in half of the chronically infected herds, the majority of isolates recovered over multiple years were ≤ 3-SNPs apart. The limited within-herd genetic diversity found recurrently in several sampled herds, on many occasions despite the considerable time elapsed between their isolations, suggests that animals chronically infected in the herd (that would have been missed in the bTB herd tests) may have contributed to disease persistence (Guta et al., 2014a). Additionally, genetic clustering of strains coming from the same herds (and same provinces) indicated that *M. bovis* was continuously circulating in the sampled herds (without introductions of new strains). Alternatively, persistence of highly similar strains may also suggest re-introductions from other sources such as neighboring herds, environmental persistence, or alternative hosts as suggested elsewhere (Biek et al., 2012).

The genetic diversity observed in the SB0339 analyzed *M. bovis* isolates was not seemingly caused by recent exogenous introductions in Spain. Instead, the observed genetic distances suggested an endemic self-maintaining infection within different animal species in each region, with certain events of interspecies transmission, which was particularly evident in the case of Madrid. Despite the small sample size included in this study, this picture is in agreement with findings of previous studies in Spain and elsewhere on the potential effect of local risk factors (i.e., spatial proximity, extensive management in beef herds, contact with other sources of infection) in bTB endemic areas (Green et al., 2008; Biek et al., 2012; Pozo et al., 2020). Additionally, our results are in agreement with a previous study conducted in Portugal, where the observed genetic diversity supported the natural circulation of *M. bovis* for a long time with multiple interactions of different host species (Reis et al., 2021). Interaction with wildlife reservoirs was identified as the second most important cause of herd breakdowns in Spain, after residual infection (Guta et al., 2014a). Indeed, *M. bovis* isolates retrieved from wildlife species in Doñana National Park were in fact more prevalent in cattle, thus contributing to bTB persistence (Romero et al., 2008). Cattle and badgers found in Northern Spain in the same geographical area and over the same period shared similar spoligotypes, suggesting common dynamics of infection (Balseiro et al., 2013). All things considered, the higher host diversity identified in the epidemiology of bTB in Spain leads to increased and more diverse transmission chains (Barasona et al., 2019). Epidemiological links across different hosts involving the same species analyzed here (i.e., wild boar and red deer) were also identified in neighboring countries such as Portugal (Cunha et al., 2012; Reis et al., 2020) and France (Hauer et al., 2015; Michelet et al., 2019), although different molecular patterns were identified. Results obtained here are of importance considering the relatively high degree of persistence that exist in several regions of Spain, such as the central and south-west parts of the country.

Sampling from different host species performed here matched over similar temporal (years) and spatial (province of origin) units. However, due to the strong dependence on isolate availability in the VISAVET strain collection and the moderate degree of success (~60%) in the re-culture of isolates, biases included in this study were unavoidable.

The potential of WGS-derived analyses may be compromised when a poorly sampled host population contributes to transmission, which in this case could be the wildlife reservoirs. Additionally, host population sampling is often limited to outbreak investigations in which sampling is reduced to the affected herds and inclusion of other sources, such as nearby farms or wildlife reservoirs, faces financial, ethical or logistical constraints. More than half of the wildlife isolates included here (*n* = 20) originated from samples collected from wild boar, mostly from Madrid, and the majority (*n* = 15/17) of the red deer and fallow deer *M. bovis* isolates were sampled in the central regions of the country as well. In contrast, other provinces were clearly underrepresented in our collection: Central Spain was more intensively sampled compared with southern regions, where bTB is highly prevalent in both cattle and wildlife (Ministerio de Agricultura, Pesca, Alimentación y Medio Ambiente, 2020; Ministerio de Agricultura, Pesca y Alimentación, 2021). Differential sampling effort across regions may have impacted the robustness of our findings, particularly regarding wildlife, when considering the whole country. Therefore, our findings should not be extrapolated to other areas unless further studies confirm the degree of diversity within and between species found here. Nevertheless, isolates belonging to the SB0339 spoligotype mostly originate from Madrid, which was the most intensively sampled region and in which a better livestock-wildlife balance was achieved (49% of the total isolates, relationship cattle:wildlife_MADRID_ 2:1 Vs. cattle:wildlife_OVERALL_ 3:1). Regarding cattle, a similar test-and-slaughter strategies based on annual testing using the single intradermal tuberculin test was in place in all provinces included here during the study period, and thus, a reasonable representation of bTB positive cattle infected with SB0339 circulating in Spain was available. Although bTB control and surveillance in wildlife is not as standardized as is in cattle and fewer wildlife isolates were available for the analysis, a high fraction of wildlife isolates were retrieved from Madrid (with both a high bTB prevalence and wildlife density), suggesting that results observed here may be a fair approximation of the actual scenario of SB0339 not only in cattle, but also in wildlife (Ministerio de Agricultura, Pesca, Alimentación y Medio Ambiente, 2020; Ministerio de Agricultura, Pesca y Alimentación, 2021).

Furthermore, among the 136 samples, there were 7 isolates with low (<20x) depth of coverage, with DNA regions of little or null coverage leading to the identification of unreliable SNPs. Likewise, the percent of the reference genome covered by these sequences was below 99%, which for clonal organisms as *M. bovis*, implies the need for a significant amount of correction. Although results obtained in these low coverage samples should be viewed with caution, no major bias was expected due to erroneous SNPs calls. These ambiguous calls occurred in non-informative SNPs (SNPs shared by all the isolates included in the study that were not relevant to define the clustering pattern presented here). Erroneous calls were meticulously verified with IGV, and reliable SNPs were manually corrected as suggested by the vSNP documentation (Orloski et al., 2018) and performed in several studies (Orloski et al., 2018; Salvador et al., 2019; Perea et al., 2021; Reis et al., 2021). In case erroneous calls fell in areas with low coverage or mapping issues these were manually filtered out.

High-throughput genotyping methods such as WGS have created unprecedented opportunities for studying the transmission network of microorganisms such as *M. bovis* and enable trace back of sources of infection, which may complement other measures included in the bTB eradication program in Spain. Given the increasing cost-effectiveness of WGS-based characterization techniques, we believe that WGS-based typing will eventually become the standard for bTB molecular epidemiological studies as has been also the trend with other pathogens (e.g., foodborne pathogens). This study confirmed that *M. bovis* is probably maintained in multi- rather than single-host populations in high but also low prevalence areas (e.g., Mallorca), and that the relative contribution of wildlife reservoirs to bTB maintenance in some regions may be low when compared to central and southwestern Spain. While traditional typing techniques have demonstrated that *M. bovis* molecular patterns are maintained within well-defined spatial clusters, their power to further discriminate strains within clusters, what could help to explain persistence and transmission, is limited (Trewby et al., 2016). In this sense, WGS results a valuable tool to improve the understanding of bTB epidemiology, even for slowly evolving and genetically conserved pathogens such as *M. bovis* (Garnier et al., 2003). Here, WGS was used to describe the genetic heterogeneity in a highly predominant spoligotype in an attempt to assess the potential for interspecies transmission irrespective of the direction, as phylogenetic trees presented here are not equivalent to transmission trees. The combination of *M. bovis* sequence data and mathematical models considering the temporal structure inherent in selected heterochronous samples may increase the statistical power to infer *M. bovis* evolutionary processes, as conducted in previous research (Glaser et al., 2016; Crispell et al., 2017, 2019; Salvador et al., 2019). The inclusion of a molecular clock into the analyses performed here and the addition of a balanced selection of samples between livestock and wildlife will be the subject of following analyses. Ultimately, the addition of the temporal scale in analysis of the genetic heterogeneities among isolates may help to quantify the role of wildlife reservoirs and livestock in *M. bovis* infection dynamics in Spain.

## Data Availability Statement

The datasets generated and analyzed for this study can be found in the National Center for Biotechnology Information Sequence Read Archive (NCBI-SRA) and can be accessed under BioProject number PRJNA804719.

## Ethics Statement

Ethical review and approval were not required for the animal study because all samples were collected as part of authorized regulatory surveillance in the framework of the Spanish National Eradication Program for Bovine Tuberculosis. Written informed consent for participation was not obtained from the owners because approval from premises owners was not required for this study.

## Author Contributions

PP and JA designed the study, wrote the manuscript, and received substantial feedback from all authors. PP and VL-L performed the bacteriological cultures of samples and DNA extraction. PP analyzed the data with the help of JA, VL-L, SR-A, JH, TS, BR, JB, and LJ. JLS provided advise on the data interpretation. All authors contributed to the article and approved the submitted version.

## Collaborators of Spanish Network on Surveillance Monitoring of Animal Tuberculosis

Members of the Spanish Network on Surveillance and Monitoring of Animal Tuberculosis that participated in this study include the Spanish Ministry of Agriculture, Fisheries and Food together with Central and Regional Laboratories; Laboratorio Central de Sanidad Animal de Santa Fe, Granada; Laboratorio Regional de Sanidad Animal, Junta de Castilla y León; Institut de Biología Animal Balears; Instituto de Investigaciones en Recursos Cinegéticos (IREC), Ciudad Real; Laboratorio Regional Agroalimentario y Ambiental de Castilla-La Mancha (LARAGA); Conselleria de Agricultura, Pesca y Alimentación, Unidad de Análisis de Sanidad Animal, Generalitat Valenciana; Departamento de Agricultura, Ganadería y Alimentación, Gobierno de Navarra; and Servicio de Ganadería y Protección Animal, Subdirección General de Higiene y Seguridad Alimentaria, Comunidad de Madrid.

## Funding

This research was a contribution to the project Integrated Strategies for Tuberculosis Control and Eradication in Spain (ERATUB; RTI2018-096010-B-C22, Ministerio de Ciencia, Innovación y Universidades). The Ministerio de Agricultura, Pesca y Alimentación, and the Área de Ganadería de la Comunidad de Madrid supported this publication. JA was the recipient of a Ramon y Cajal contract from the MINECO (RYC-2016-20422).

## Conflict of Interest

The authors declare that the research was conducted in the absence of any commercial or financial relationships that could be construed as a potential conflict of interest.

## Publisher’s Note

All claims expressed in this article are solely those of the authors and do not necessarily represent those of their affiliated organizations, or those of the publisher, the editors and the reviewers. Any product that may be evaluated in this article, or claim that may be made by its manufacturer, is not guaranteed or endorsed by the publisher.

## Footnotes

^1^ dx.doi.org/10.17504/protocols.io.nsgdebw

^2^ https://github.com/USDA-VS/vSNP
