# Supplementary material for: Use of Whole-Genome Sequencing to Unravel the Genetic Diversity of a Prevalent Mycobacterium bovis Spoligotype in a Multi-Host Scenario in Spain
Source: Front Microbiol. 2022 Jul 11;13:915843. doi: 10.3389/fmicb.2022.915843 (PMC9309649; doi:10.3389/fmicb.2022.915843)
Supplement: Supplementary file 1 [file Data_Sheet_1.PDF]

Figure S1. Number of *M. bovis* SB0339 isolates recovered from cattle and wildlife per year included in the study.

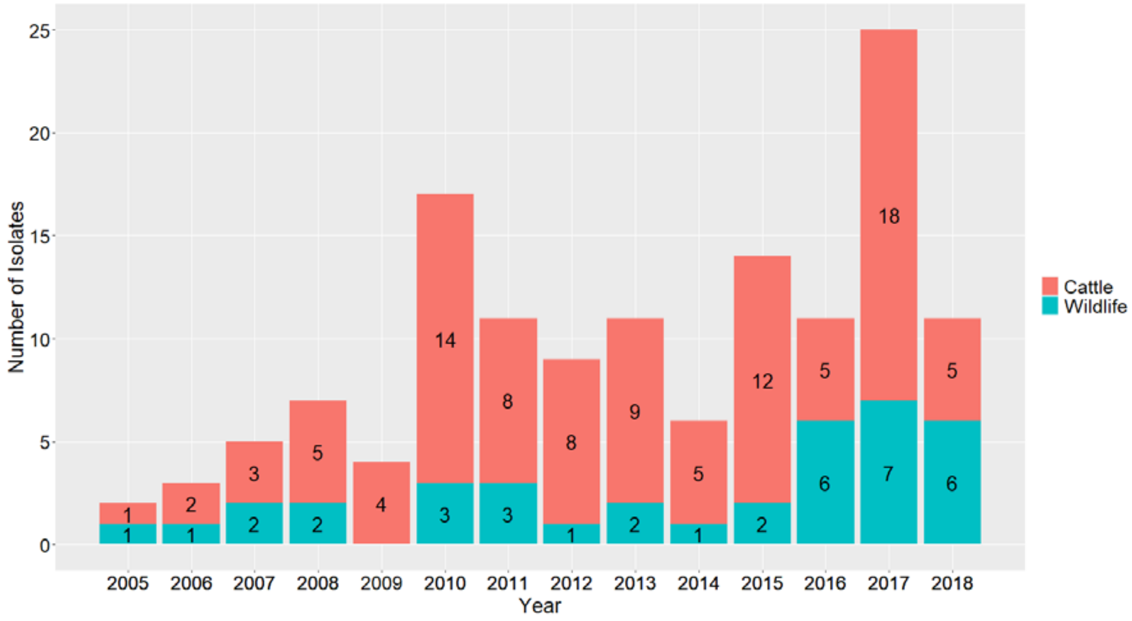

Figure S2. Whole genome sequence RAxML phylogenetic trees constructed using a GTR-CAT model of 136 Spanish *M. bovis* SB0339 samples. Colors signal different clades. Numbers below the branches denote bootstrap support values.

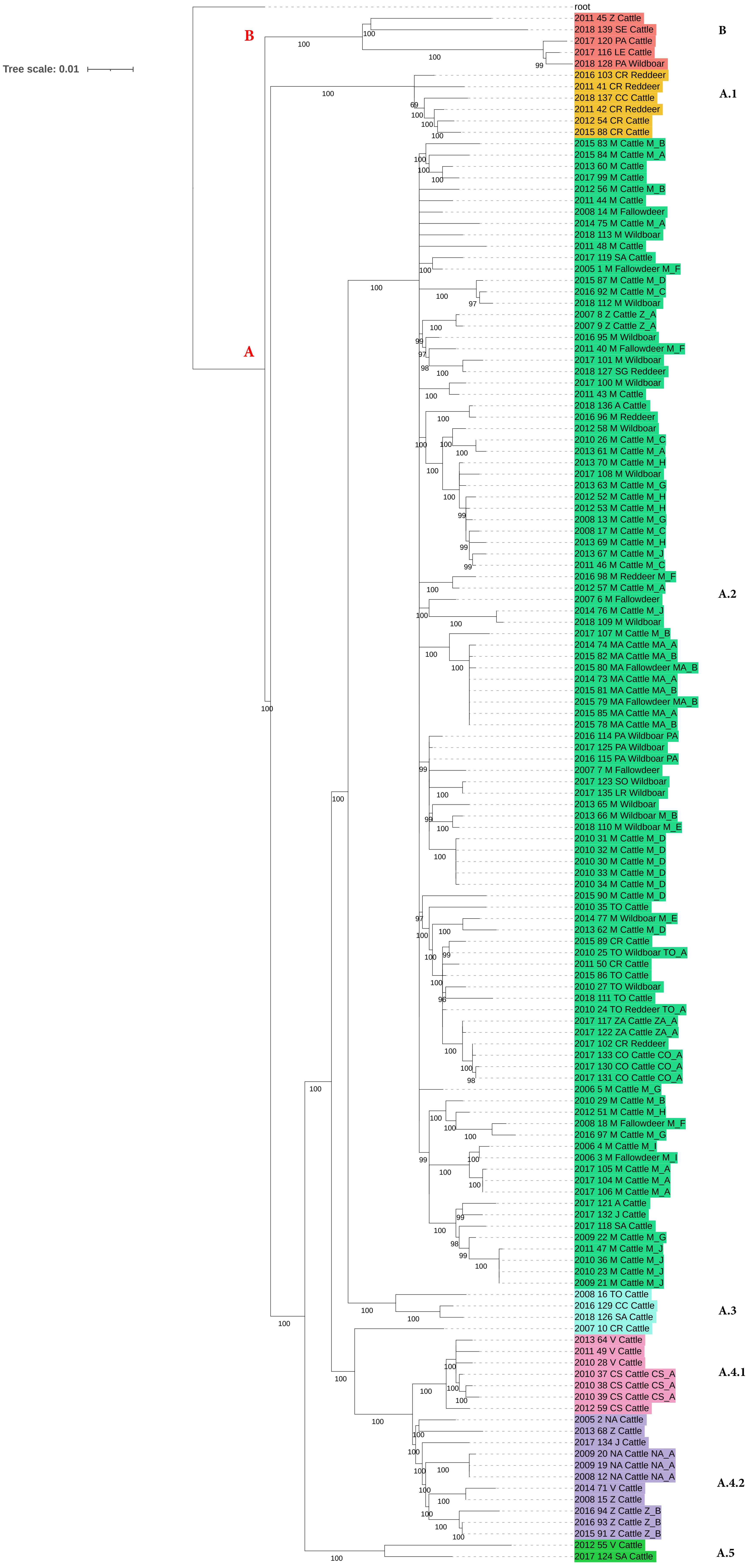

Figure S3. Whole genome sequence RAxML phylogenetic tree constructed using a GTR-CAT model of 136 Spanish *M. bovis* SB0339 samples. Colors signal different clades annotated with the second level clusters identified using rhierBAPS.

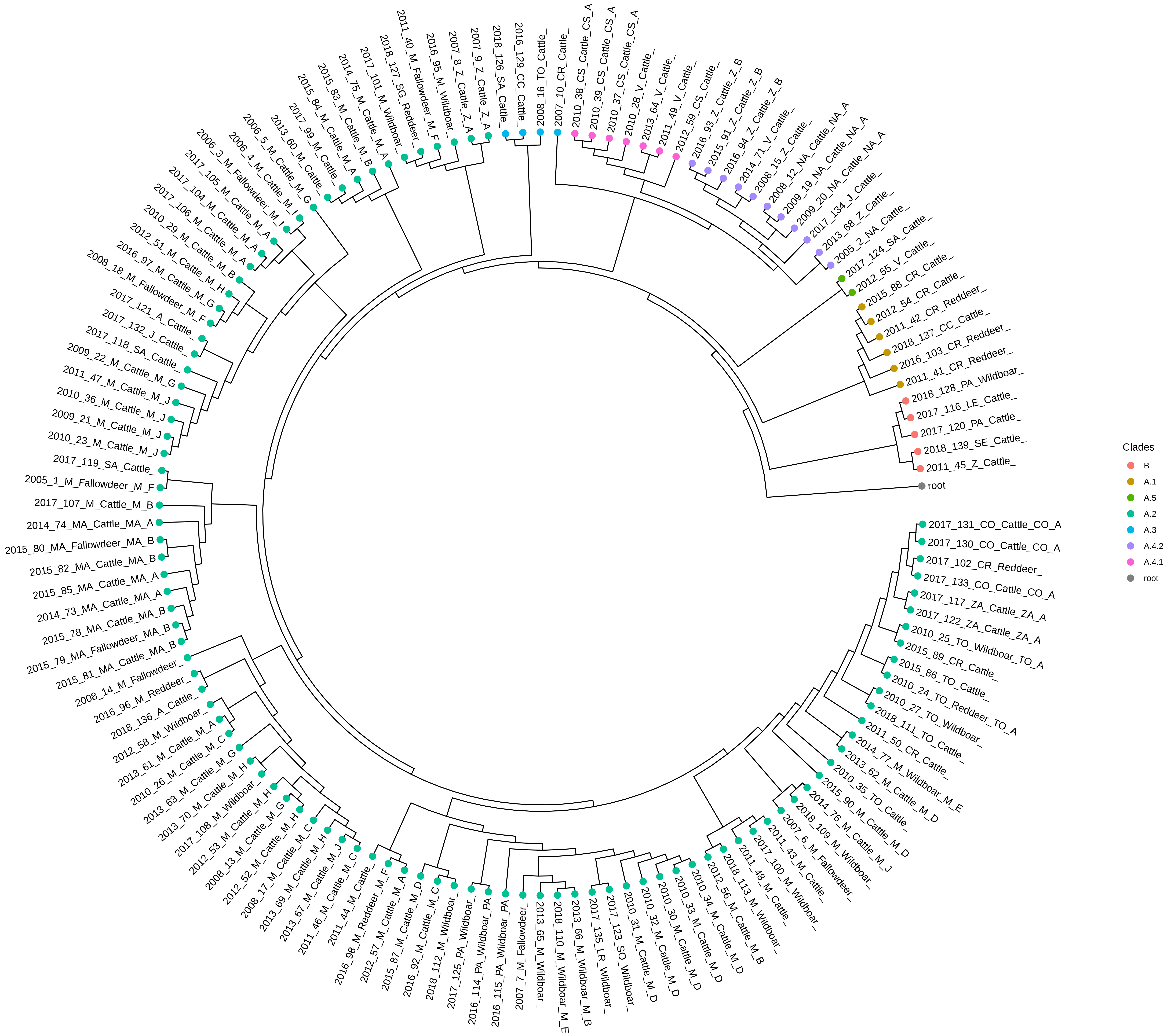

Figure S4. Distribution of clades per province identified in the study. Colors denote diferent clades.

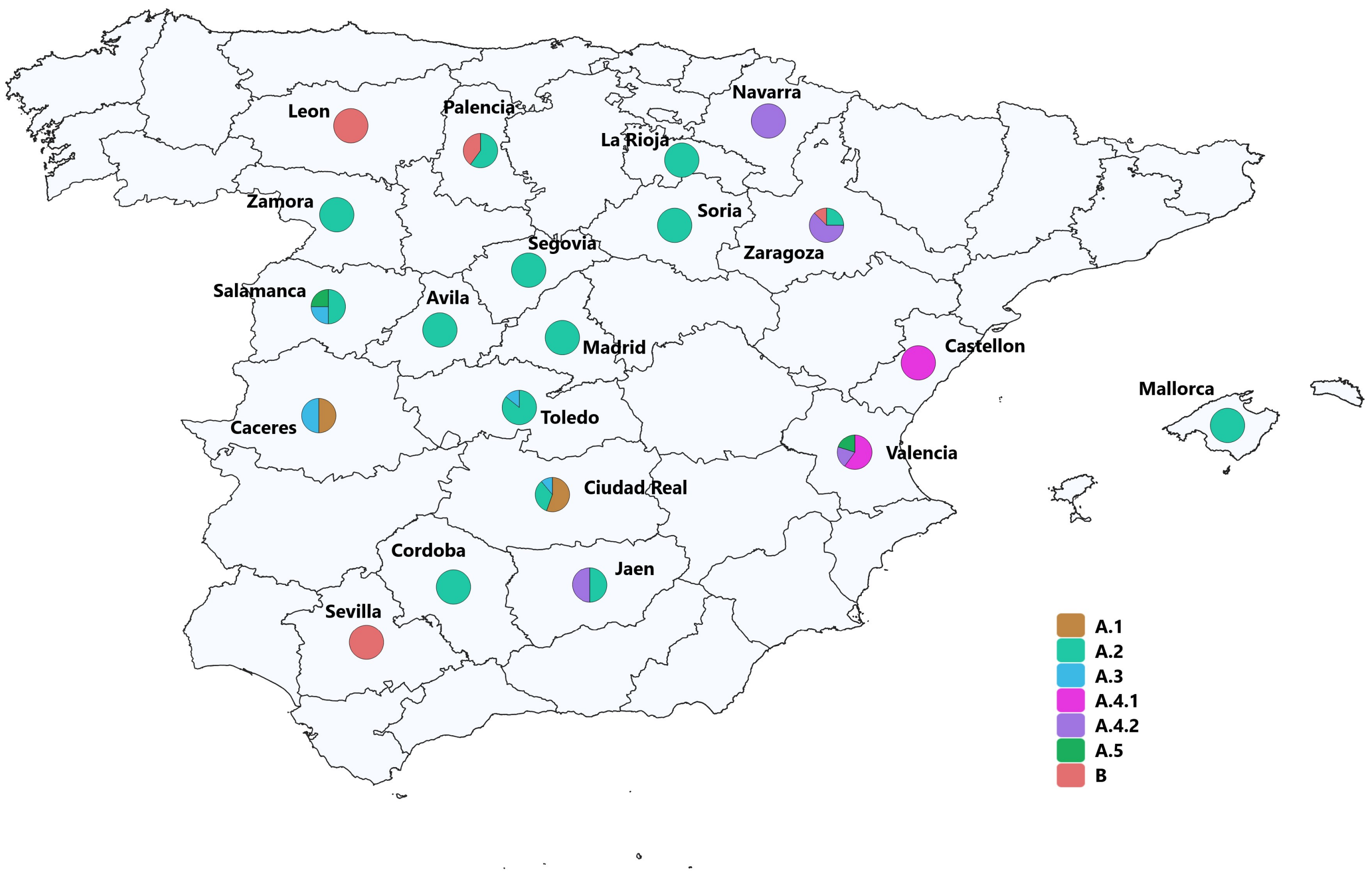

Figure S5. Analysis of pairwise genetic distances between isolates recovered within- and between-species as a function of their geographic distances of isolation in the remaining 9 replicas based on random selection of one isolate per chronically infected herd. Linear regression lines were fitted to denote the relationship between genetic and geographic distances per animal species combination and formulas were depicted for each category. Colors denote different combinations of pairs of isolates retrieved from cattle (red), wildlife (green) and collected from cattle and wildlife (blue).

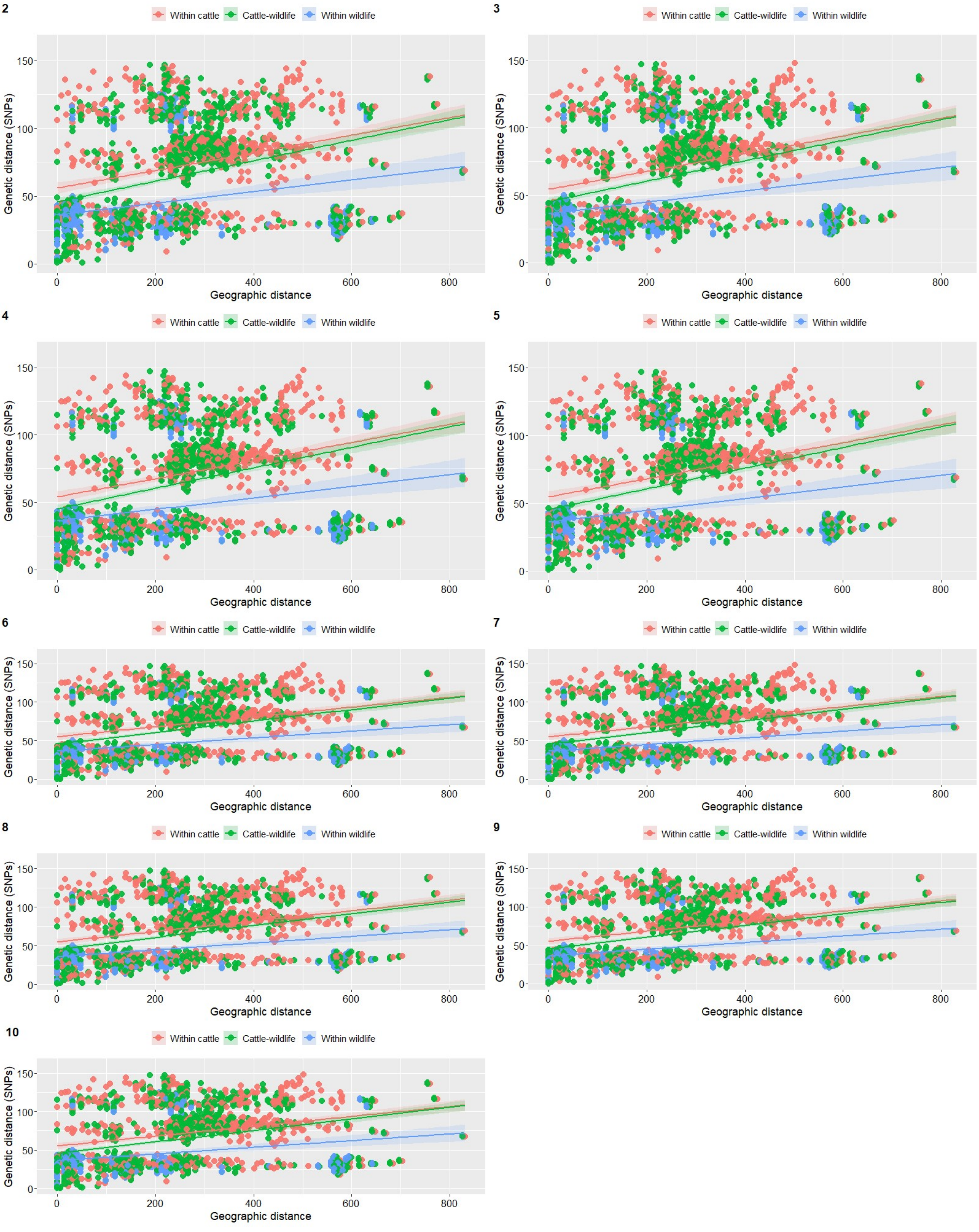

Figure S6. Whole genome sequence RAxML phylogenetic tree constructed using a GTR-CAT model of 61 *M. bovis* SB0339 cattle samples recovered from the 15 herds for the analysis of the within-herd genomic diversity. Tips are colored based on the province of isolation.

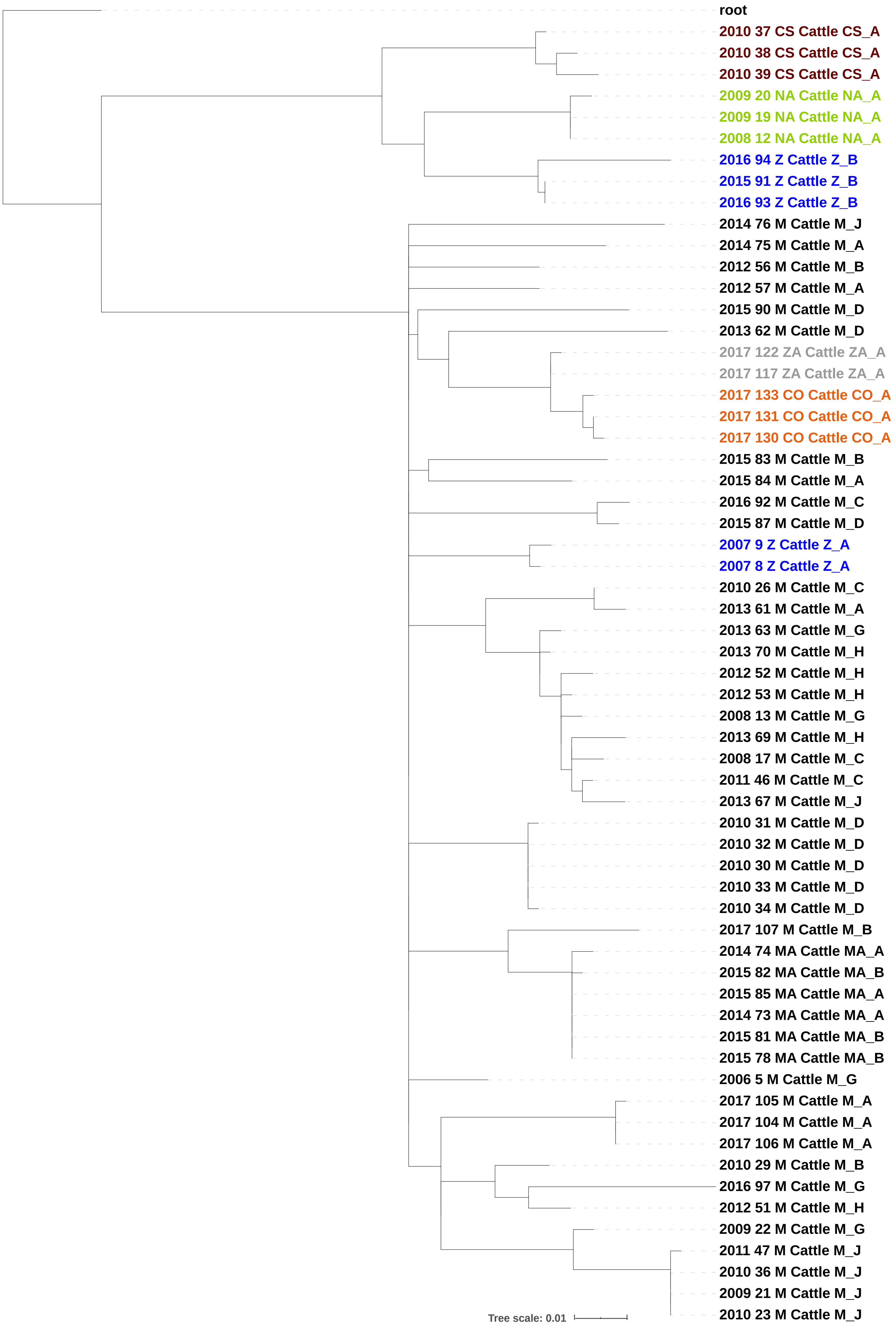

Table S1. Isolate information and associated metadata of the 136 samples included in the study. Municipality of origin for Madrid isolates is denoted with letters at the bottom of the table.

Isolate ID	Reference ID	Animal species	Province/ region	Herd/reserve	Year of isolation	Genome coverage (%)	Average coverage (x)	Average read length	Unmapped assembled contigs	Good SNP count	Octalcode	Spoligotype
1	2005_1_M_Fallow deer_MF[b]	Fallow deer	Madrid	M_F	2005	98.72	24.2	218.8	1	612	676773674177600	SB0339
2	2005_2_NA_Cattle	Cattle	Navarra		2005	99.04	93.7	230.8	1	653	676773674177600	SB0339
3	2006_3_M_Fallow deer_MI[a]	Fallow deer	Madrid	M_I	2006	98.89	49.0	225.4	3	647	676773674177600	SB0339
4	2006_4_M_Cattle_MI[a]	Cattle	Madrid	M_I	2006	98.86	41.9	229.3	1	634	676773674177600	SB0339
5	2006_5_M_Cattle_MG[a]	Cattle	Madrid	M_G	2006	96.66	7.9	186.5	0	411	676772214170600	SB0339
6	2007_6_M_Fallow deer[b]	Fallow deer	Madrid		2007	98.84	33.1	218.7	1	620	676773674177600	SB0339
7	2007_7_M_Fallow deer[b]	Fallow deer	Madrid		2007	98.89	42.1	221.9	18	629	676773674177600	SB0339
8	2007_8_Z_Cattle_ZA	Cattle	Zaragoza	Z_A	2007	98.78	24.0	216.2	1	611	676773674177600	SB0339
9	2007_9_Z_Cattle_ZA	Cattle	Zaragoza	Z_A	2007	98.90	57.6	223.7	20	634	676773674177600	SB0339
10	2007_10_CR_Cattle	Cattle	Ciudad Real		2007	99.05	64.5	222.7	2	644	676773674177600	SB0339
12	2008_12_NA_Cattle_NAA	Cattle	Navarra	NA_A	2008	98.99	68.4	228.1	446	875	676773674177600	SB0339
13	2008_13_M_Cattle_MG[a]	Cattle	Madrid	M_G	2008	99.06	84.7	229.1	2	650	676773674177600	SB0339
14	2008_14_M_Fallow deer[b]	Fallow deer	Madrid		2008	99.06	68.5	229.5	2	640	676773674177600	SB0339
15	2008_15_Z_Cattle	Cattle	Zaragoza		2008	98.85	54.2	230.3	4	648	676773674177600	SB0339
16	2008_16_TO_Cattle	Cattle	Toledo		2008	98.78	29.8	217.1	5	623	676773674177600	SB0339
17	2008_17_M_Cattle_MC[a]	Cattle	Madrid	M_C	2008	99.04	90.2	228.0	2	649	676773674177600	SB0339
18	2008_18_M_Fallow deer_MF[b]	Fallow deer	Madrid	M_F	2008	98.93	87.7	233.1	5	658	676773674177600	SB0339
19	2009_19_NA_Cattle_NAA	Cattle	Navarra	NA_A	2009	99.07	67.3	212.4	46	654	676773674177600	SB0339
20	2009_20_NA_Cattle_NAA	Cattle	Navarra	NA_A	2009	98.84	21.3	199.6	1	614	676773674177600	SB0339
21	2009_21_M_Cattle_MJ[f]	Cattle	Madrid	M_J	2009	99.04	100.8	230.2	1	665	676773674177600	SB0339

22	2009_22_M_Cattle_MG[a]	Cattle	Madrid	M_G	2009	99.04	93.0	229.3	1	651	676773674177600	SB0339
23	2010_23_M_Cattle_MJ[f]	Cattle	Madrid	M_J	2010	99.06	106.6	228.2	1	664	676773674177600	SB0339
24	2010_24_TO_Red deer_TOA	Red deer	Toledo	TO_A	2010	97.93	9.7	215.3	745	562	676773674177600	SB0339
25	2010_25_TO_Wild boar_TOA	Wild boar	Toledo	TO_A	2010	98.60	15.3	220.2	726	641	676773674177600	SB0339
26	2010_26_M_Cattle_MC[a]	Cattle	Madrid	M_C	2010	99.00	91.1	238.0	1	659	676773674177600	SB0339
27	2010_27_TO_Wild boar	Wild boar	Toledo		2010	98.82	27.0	214.9	1	622	676773674177600	SB0339
28	2010_28_V_Cattle	Cattle	Valencia		2010	99.04	95.9	233.1	14	646	676773674177600	SB0339
29	2010_29_M_Cattle_MB[a]	Cattle	Madrid	M_B	2010	98.79	77.5	237.4	1	645	676773674177600	SB0339
30	2010_30_M_Cattle_MD[b]	Cattle	Madrid	M_D	2010	99.04	92.2	226.6	3	655	676773674177600	SB0339
31	2010_31_M_Cattle_MD[b]	Cattle	Madrid	M_D	2010	99.04	108.4	234.3	19	662	676773674177600	SB0339
32	2010_32_M_Cattle_MD[b]	Cattle	Madrid	M_D	2010	99.01	96.7	233.2	125	648	676773674177600	SB0339
33	2010_33_M_Cattle_MD[b]	Cattle	Madrid	M_D	2010	98.37	12.7	196.5	45	552	676773674177600	SB0339
34	2010_34_M_Cattle_MD[b]	Cattle	Madrid	M_D	2010	98.42	18.1	229.0	4	598	674773674177600	SB0339
35	2010_35_TO_Cattle	Cattle	Toledo		2010	99.04	100.8	234.1	1	652	676773674177600	SB0339
36	2010_36_M_Cattle_MJ[f]	Cattle	Madrid	M_J	2010	98.91	74.8	236.2	1	666	676773674177600	SB0339
37	2010_37_CS_Cattle_CSA	Cattle	Castellon	CS_A	2010	98.74	30.6	219.6	17	634	676773674177600	SB0339
38	2010_38_CS_Cattle_CSA	Cattle	Castellon	CS_A	2010	99.09	152.2	234.3	55	669	676773674177600	SB0339
39	2010_39_CS_Cattle_CSA	Cattle	Castellon	CS_A	2010	99.01	93.5	237.7	8	662	676773674177600	SB0339
40	2011_40_M_Fallow deer_MF[b]	Fallow deer	Madrid	M_F	2011	99.00	88.0	237.6	1	643	676773674177600	SB0339
41	2011_41_CR_Red deer_	Red deer	Ciudad Real		2011	98.72	22.6	212.5	9	606	676773674177600	SB0339
42	2011_42_CR_Red deer_	Red deer	Ciudad Real		2011	98.63	14.0	228.1	1603	675	676773674177600	SB0339
43	2011_43_M_Cattle[a]	Cattle	Madrid		2011	99.02	76.9	235.7	24	646	676773674177600	SB0339
44	2011_44_M_Cattle[a]	Cattle	Madrid		2011	98.98	74.7	236.4	171	658	676773674177600	SB0339
45	2011_45_Z_Cattle	Cattle	Zaragoza		2011	98.79	29.8	218.9	1	624	676773674177600	SB0339
46	2011_46_M_Cattle_MC[a]	Cattle	Madrid	M_C	2011	98.96	73.8	235.5	2	645	676773674177600	SB0339
47	2011_47_M_Cattle_MJ[f]	Cattle	Madrid	M_J	2011	98.98	89.0	234.5	2	664	676773674177600	SB0339

48	2011_48_M_Cattle[a]	Cattle	Madrid		2011	99.00	71.2	233.5	130	652	676773674177600	SB0339
49	2011_49_V_Cattle	Cattle	Valencia		2011	98.93	70.6	235.5	1	658	676773674177600	SB0339
50	2011_50_CR_Cattle	Cattle	Ciudad Real		2011	99.12	126.2	223.4	5	662	676773674177600	SB0339
51	2012_51_M_Cattle_MH[b]	Cattle	Madrid	M_H	2012	98.36	23.4	214.3	1	613	676773674177600	SB0339
52	2012_52_M_Cattle_MH[b]	Cattle	Madrid	M_H	2012	98.99	69.5	233.5	1	640	676773674177600	SB0339
53	2012_53_M_Cattle_MH[b]	Cattle	Madrid	M_H	2012	98.00	7.5	228.2	3072	500	676773674177600	SB0339
54	2012_54_CR_Cattle	Cattle	Ciudad Real		2012	98.91	57.5	235.3	5265	626	676773674177600	SB0339
55	2012_55_V_Cattle	Cattle	Valencia		2012	98.96	70.1	236.9	13	664	676773674177600	SB0339
56	2012_56_M_Cattle_MB[a]	Cattle	Madrid	M_B	2012	99.10	131.6	231.0	1	652	676773674177600	SB0339
57	2012_57_M_Cattle_MA[a]	Cattle	Madrid	M_A	2012	99.10	100.9	231.5	1	648	676773674177600	SB0339
58	2012_58_M_Wild boar[g]	Wild boar	Madrid		2012	99.06	103.4	231.0	1	663	676773674177600	SB0339
59	2012_59_CS_Cattle	Cattle	Castellon		2012	98.59	26.6	236.0	1	611	676773674177600	SB0339
60	2013_60_M_Cattle[a]	Cattle	Madrid		2013	98.74	46.7	226.4	4	628	676773674177600	SB0339
61	2013_61_M_Cattle_MA[a]	Cattle	Madrid	M_A	2013	99.11	146.8	228.8	1	670	676773674177600	SB0339
62	2013_62_M_Cattle_MD[b]	Cattle	Madrid	M_D	2013	99.04	90.5	227.4	1	655	676773674177600	SB0339
63	2013_63_M_Cattle_MG[a]	Cattle	Madrid	M_G	2013	98.92	42.4	219.6	1	630	676773674177600	SB0339
64	2013_64_V_Cattle	Cattle	Valencia		2013	99.04	75.9	231.3	1	658	676773674177600	SB0339
65	2013_65_M_Wild boar[a]	Wild boar	Madrid		2013	98.92	57.6	223.3	1	650	676773674177600	SB0339
66	2013_66_M_Wild boar_MB[a]	Wild boar	Madrid	M_B	2013	98.97	76.5	233.7	3	649	676773674177600	SB0339
67	2013_67_M_Cattle_MJ[f]	Cattle	Madrid	M_J	2013	98.90	47.8	239.4	1	645	676773674177600	SB0339
68	2013_68_Z_Cattle	Cattle	Zaragoza		2013	99.04	85.1	226.5	1472	665	676773674177600	SB0339
69	2013_69_M_Cattle_MH[b]	Cattle	Madrid	M_H	2013	98.74	24.0	212.5	12	622	676773674177600	SB0339
70	2013_70_M_Cattle_MH[b]	Cattle	Madrid	M_H	2013	98.98	55.9	219.2	1	636	676773674177600	SB0339
71	2014_71_V_Cattle	Cattle	Valencia		2014	99.02	98.3	232.4	788	661	676773674177600	SB0339
73	2014_73_MA_Cattle_MAA	Cattle	Mallorca	MA_A	2014	99.07	102.3	231.5	1	676	676773674177600	SB0339
74	2014_74_MA_Cattle_MAA	Cattle	Mallorca	MA_A	2014	99.05	97.2	230.6	33	680	676773674177600	SB0339

75	2014_75_M_Cattle_MA[a]	Cattle	Madrid	M_A	2014	98.91	54.0	235.4	1	645	676773674177600	SB0339
76	2014_76_M_Cattle_MJ[f]	Cattle	Madrid	M_J	2014	99.06	119.9	231.4	1	673	676773674177600	SB0339
77	2014_77_M_Wild boar_ME[a]	Wild boar	Madrid	M_E	2014	98.85	27.0	210.8	3	621	676773674177600	SB0339
78	2015_78_MA_Cattle_MAB	Cattle	Mallorca	MA_B	2015	99.04	81.7	234.5	2	659	676773674177600	SB0339
79	2015_79_MA_Fallow deer_MAB	Fallow deer	Mallorca	MA_B	2015	99.08	120.1	228.7	1716	701	676773674177600	SB0339
80	2015_80_MA_Fallow deer_MAB	Fallow deer	Mallorca	MA_B	2015	98.83	44.8	237.5	1	643	676773674177600	SB0339
81	2015_81_MA_Cattle_MAB	Cattle	Mallorca	MA_B	2015	99.11	134.0	232.5	1	681	676773674177600	SB0339
82	2015_82_MA_Cattle_MAB	Cattle	Mallorca	MA_B	2015	98.81	41.3	234.5	1	645	676773674177600	SB0339
83	2015_83_M_Cattle_MB[a]	Cattle	Madrid	M_B	2015	99.08	110.9	230.2	1	662	676773674177600	SB0339
84	2015_84_M_Cattle_MA[a]	Cattle	Madrid	M_A	2015	98.92	40.6	214.2	8	637	676773674177600	SB0339
85	2015_85_MA_Cattle_MAA	Cattle	Mallorca	MA_A	2015	99.04	88.4	235.8	1	666	676773674177600	SB0339
86	2015_86_TO_Cattle	Cattle	Toledo		2015	98.74	35.6	230.5	1	621	676773674177600	SB0339
87	2015_87_M_Cattle_MD[b]	Cattle	Madrid	M_D	2015	98.79	29.6	210.5	2	621	676773674177600	SB0339
88	2015_88_CR_Cattle	Cattle	Ciudad Real		2015	98.84	36.8	214.0	2	626	676773674177600	SB0339
89	2015_89_CR_Cattle	Cattle	Ciudad Real		2015	99.08	107.8	227.3	1	655	676773674177600	SB0339
90	2015_90_M_Cattle_MD[b]	Cattle	Madrid	M_D	2015	99.08	107.5	229.9	5	663	676773674177600	SB0339
91	2015_91_Z_Cattle_ZB	Cattle	Zaragoza	Z_B	2015	99.04	81.7	224.9	5320	869	676773674177600	SB0339
92	2016_92_M_Cattle_MC[a]	Cattle	Madrid	M_C	2016	99.06	98.8	230.3	2	664	676773674177600	SB0339
93	2016_93_Z_Cattle_ZB	Cattle	Zaragoza	Z_B	2016	98.84	33.4	216.5	1	622	676773674177600	SB0339
94	2016_94_Z_Cattle_ZB	Cattle	Zaragoza	Z_B	2016	98.69	23.6	210.1	1	622	676773674177600	SB0339
95	2016_95_M_Wild boar[a]	Wild boar	Madrid		2016	98.94	75.7	231.9	4	647	676773674177600	SB0339
96	2016_96_M_Red deer[b]	Red deer	Madrid		2016	98.84	37.5	225.9	1	627	676773674177600	SB0339
97	2016_97_M_Cattle_MG[a]	Cattle	Madrid	M_G	2016	98.87	99.7	234.9	2	680	676773674177600	SB0339
98	2016_98_M_Red deer_MF[b]	Red deer	Madrid	M_F	2016	99.03	54.6	230.3	8	645	676773674177600	SB0339
99	2017_99_M_Cattle[a]	Cattle	Madrid		2017	99.01	80.4	231.3	6	662	676773674177600	SB0339
100	2017_100_M_Wild boar[a]	Wild boar	Madrid		2017	99.08	125.6	233.6	1	660	676773674177600	SB0339

101	2017_101_M_Wild boar[a]	Wild boar	Madrid		2017	98.92	50.7	222.1	1	640	676773674177600	SB0339
102	2017_102_CR_Red deer	Red deer	Ciudad Real		2017	99.08	96.0	228.8	5	658	676773674177600	SB0339
103	2016_103_CR_Red deer	Red deer	Ciudad Real		2016	98.99	98.1	235.4	2	647	676773674177600	SB0339
104	2017_104_M_Cattle_MA[a]	Cattle	Madrid	M_A	2017	98.78	30.0	212.9	877	658	676773674177600	SB0339
105	2017_105_M_Cattle_MA[a]	Cattle	Madrid	M_A	2017	99.06	95.5	230.0	1	677	676773674177600	SB0339
106	2017_106_M_Cattle_MA[a]	Cattle	Madrid	M_A	2017	98.88	34.6	204.4	1	630	676773674177600	SB0339
107	2017_107_M_Cattle_MB[a]	Cattle	Madrid	M_B	2017	99.08	116.0	230.9	1	668	676773674177600	SB0339
108	2017_108_M_Wild boar[g]	Wild boar	Madrid		2017	99.07	97.1	231.1	3	656	676773674177600	SB0339
109	2018_109_M_Wild boar[d]	Wild boar	Madrid		2018	99.08	86.5	221.6	9	676	676773674177600	SB0339
110	2018_110_M_Wild boar_ME[a]	Wild boar	Madrid	M_E	2018	98.98	45.3	213.5	1	644	676773674177600	SB0339
111	2018_111_TO_Cattle	Cattle	Toledo		2018	99.27	49.2	227.1	1	643	676773674177600	SB0339
112	2018_112_M_Wild boar[c]	Wild boar	Madrid		2018	99.01	100.9	239.1	1	666	676773674177600	SB0339
113	2018_113_M_Wild boar[a]	Wild boar	Madrid		2018	99.14	94.8	220.1	1099	890	676773674177600	SB0339
114	2016_114_PA_Wild boar_PA	Wild boar	Palencia	P_A	2016	99.16	117.6	225.5	1	645	676773674177600	SB0339
115	2016_115_PA_Wild boar_PA	Wild boar	Palencia	P_A	2016	99.12	137.1	228.5	2	655	676773674177600	SB0339
116	2017_116_LE_Cattle	Cattle	Leon		2017	99.00	83.1	236.0	1	672	676773674177600	SB0339
117	2017_117_ZA_Cattle_ZAA	Cattle	Zamora	ZA_A	2017	99.10	113.2	228.2	1	667	676773674177600	SB0339
118	2017_118_SA_Cattle	Cattle	Salamanca		2017	99.07	87.0	231.4	1	654	676773674177600	SB0339
119	2017_119_SA_Cattle	Cattle	Salamanca		2017	99.07	87.0	239.0	1	648	676773674177600	SB0339
120	2017_120_PA_Cattle	Cattle	Palencia		2017	99.03	92.6	236.4	1	672	676773674177600	SB0339
121	2017_121_A_Cattle	Cattle	Avila		2017	98.99	76.3	234.9	2	659	676773674177600	SB0339
122	2017_122_ZA_Cattle_ZAA	Cattle	Zamora	ZA_A	2017	99.22	173.9	206.2	3304	840	676773674177600	SB0339
123	2017_123_SO_Wild boar	Wild boar	Soria		2017	99.02	84.8	234.4	2	644	676773674177600	SB0339
124	2017_124_SA_Cattle	Cattle	Salamanca		2017	98.99	105.0	236.4	1	654	676773674177600	SB0339
125	2017_125_PA_Wild boar	Wild boar	Palencia		2017	99.00	99.0	238.4	1	641	676773674177600	SB0339
126	2018_126_SA_Cattle	Cattle	Salamanca		2018	99.04	111.3	236.8	1	646	676773674177600	SB0339

127	2018_127_SG_Red deer	Red deer	Segovia		2018	99.06	101.8	233.4	1	651	676773674177600	SB0339
128	2018_128_PA_Wild boar	Wild boar	Palencia		2018	99.12	89.7	219.0	894	701	676773674177600	SB0339
129	2016_129_CC_Cattle	Cattle	Caceres		2016	99.07	117.2	229.0	3	653	676773674177600	SB0339
130	2017_130_CO_Cattle_COA	Cattle	Cordoba	CO_A	2017	99.11	120.0	228.7	3	660	676773674177600	SB0339
131	2017_131_CO_Cattle_COA	Cattle	Cordoba	CO_A	2017	99.06	108.5	231.5	4	652	676773674177600	SB0339
132	2017_132_J_Cattle	Cattle	Jaen		2017	98.97	107.3	232.1	2	661	676773674177600	SB0339
133	2017_133_CO_Cattle_COA	Cattle	Cordoba	CO_A	2017	99.03	110.0	231.5	5	647	676773674177600	SB0339
134	2017_134_J_Cattle	Cattle	Jaen		2017	99.08	127.1	225.5	4	661	676773674177600	SB0339
135	2017_135_LR_Wild boar	Wild boar	La Rioja		2017	99.06	119.0	228.8	3	652	676773674177600	SB0339
136	2018_136_A_Cattle	Cattle	Avila		2018	99.09	120.5	230.3	2	665	676773674177600	SB0339
137	2018_137_CC_Cattle	Cattle	Caceres		2018	99.13	125.1	222.0	1	650	676773674177600	SB0339
139	2018_139_SE_Cattle	Cattle	Sevilla		2018	98.84	104.3	226.0	1	670	676773674177600	SB0339

[a] Municipality 1; [b] Municipality 2; [c] Municipality 3; [d] Municipality 4; [e] Municipality 5; [f] Municipality 6; [g] Municipality 7

Table S2. Information on clades, number of isolates and median distances with interquartile ranges (IQR) and ranges within- and between-clades.

Group	Clade	Number of isolates	Combination of groups/clades	Median distance (IQR)	Range
**A**		**131**<br>95 cattle<br>36 wildlife	**A - A**	**37 (29-82)**	**0-129**
	**A1**	**6**<br>3 cattle<br>3 wildlife	**A1 – A1**	**21 (17-24)**	**9-31**
			A1 – A2	113 (108-117)	94-129
			A1 – A3	108 (105-111)	98-115
			A1 – A4.1	114 (110-116)	104-119
			A1-A4.2	114 (109-116)	101-125
			A1-A5	114 (108-124)	100-127
	**A2**	**101**<br>68 cattle<br>33 wildlife	**A2 – A2**	**30 (25-35)**	**0-52**
			A2 – A3	71 (67-75)	55-87
			A2 – A4.1	83 (80-87)	68-98
			A2 – A4.2	83 (79-88)	65-103
			A2 – A5	101 (92-109)	80-122
	**A3**	**4** cattle	**A3 – A3**	**55 (39-71)**	**11-73**
			A3 – A4.1	80 (72-81)	58-84
			A3 – A4.2	79 (71-81)	55-90
			A3 – A5	96 (89-105)	84-108
	**A4.1**	**7** cattle	A4.1 – A4.1	12 (9-14)	5-17
			A4.1 – A4.2	35 (33-39)	27-46
			A4.1 – A5	101 (93-110)	90-112
	**A4.2**	**11** cattle	**A4.2 – A4.2**	**28 (25-35)**	**0-45**
			A4.2 – A5	103 (92-109)	87-118
	**A5**	**2** cattle	**A5 – A5**	**57**	**57**
**B**	**B**	**5**<br>4 cattle<br>1 wildlife	**B - B**	**96 (32-103)**	**12-108**
			A1 – B	138 (127-144)	115-148
			A2 - B	144 (135-150)	115-163
			A3 – B	141 (131-145)	119-149
			A4.1 – B	147 (136-149)	125-153
			A4.2 – B	145 (135-150)	122-159
			A5 – B	143 (139-155)	121-161
**A - B**				144 (135-149)	115-163

Table S3. Results from the 10 multivariable linear regression models performed by random selection of one isolate per chronically infected herd to evaluate the association between genetic and geographic distances considering the host from which isolates originated.

Variable	Levels	Random sample1		Random sample2		Random sample3		Random sample4		Random sample5	
		**Coefficient (95% CI)**	***P* - value**	**Coefficient (95% CI)**	***P* - value**	**Coefficient (95% CI)**	***P* - value**	**Coefficient (95% CI)**	***P* - value**	**Coefficient (95% CI)**	***P* - value**
Intercept		55.02 (50.9-59.1)	<0.001	54.73 (50.7-58.8)	<0.001	55.25 (51.2-59.3)	<0.001	55.15 (51.1-59.2)	<0.001	55.98 (51.9-60.0)	<0.001
Geographic distance (km)		0.07 (<0.1-0.1)	<0.001	0.07 (<0.1-<0.1)	<0.001	0.07 (<0.1-0.1)	<0.001	0.07 (<0.1-0.1)	<0.001	0.06 (<0.1-<0.1)	<0.001
Animal species	Cattle-wildlife	-9.56 (-14.6- -4.5)	<0.001	-9.26 (-14.3- -4.3)	<0.001	-9.40 (-14.4- -4.4)	<0.001	-9.42 (-14.4- -4.4)	<0.001	-10.10 (-15.1- -5.1)	<0.001
	Within wildlife	-18.88 (-24.6- -13.2)	<0.001	-18.60 (-24.5- -12.9)	<0.001	-19.1 (-24.8- -13.4)	<0.001	-19.01 (-24.7- -13.3)	<0.001	-19.85 (-25.6- -14.1)	<0.001
Geographic distance: Cattle-wildlife		0.01 (<-0.1-<0.1)	0.230	0.01 (<-0.1- <0.1)	0.273	0.01 (<-0.1-<0.1)	0.254	0.01 (<-0.1-<0.1)	0.248	0.01 (<-0.1-<0.1)	0.187
Geographic distance: Within-wildlife		-0.02 (<-0.1-<-0.1)	0.033	-0.02 (<-0.1- <-0.1)	0.027	-0.02 (<-0.1- <-0.1)	0.039	-0.02 (<-0.1-<-0.1)	0.037	-0.02 (<-0.1-<-0.1)	0.041

Variable	Levels	Random sample6		Random sample7		Random sample8		Random sample9		Random sample10	
		**Coefficient (95% CI)**	***P* - value**	**Coefficient (95% CI)**	***P* - value**	**Coefficient (95% CI)**	***P* - value**	**Coefficient (95% CI)**	***P* - value**	**Coefficient (95% CI)**	***P* - value**
Intercept		55.09 (51.0-59.1)	<0.001	53.90 (49.9-57.9)	<0.001	54.72 (50.7-58.8)	<0.001	55.63 (51.6-59.7)	<0.001	55.72 (51.7-59.7)	<0.001
Geographic distance (km)		0.07 (0.1-0.1)	<0.001	0.07 (0.1-0.1)	<0.001	0.07 (<0.1-0.1)	<0.001	0.07 (<0.1-0.1)	<0.001	0.07 (<0.1-0.1)	<0.001
Animal species	Cattle-wildlife	-9.49 (-14.5- -4.5)	<0.001	-8.74 (-13.8- -3.8)	<0.001	-9.54 (-14.6- -4.5)	<0.001	-9.91 (-14.9- -4.9)	<0.001	-9.66 (-14.7- -4.7)	<0.001
	Within wildlife	-18.95 (-24.7- -13.2)	<0.001	-17.76 (-23.5- -12.1)	<0.001	-18.58 (-24.3- -12.9)	<0.001	-19.49 (-25.2- -13.8)	<0.001	-19.58 (-25.3- -13.9)	<0.001
Geographic distance: Cattle-wildlife		0.01 (<-0.1-<0.1)	0.250	0.01 (<-0.1-<0.1)	0.302	0.01 (<-0.1-0.1)	0.229	0.01 (<-0.1-0.1)	0.217	0.01 (<-0.1-0.1)	0.239
Geographic distance: Within-wildlife		-0.02 (<-0.1-<0.1)	0.031	-0.02 (<-0.1-<-0.1)	0.025	-0.02 (<-0.1-<-0.1)	0.026	-0.02 (<-0.1-<-0.1)	0.038	-0.02 (<-0.1-<0.1)	0.050

Table S4. Results from the 10 multivariable linear regression models performed by random selection of one isolate per chronically infected herd in isolates recovered in Madrid.

Variable	Random sample1		Random sample2		Random sample3		Random sample4		Random sample5	
	Coefficient (95% CI)	*P* -value	Coefficient (95% CI)	*P* -value	Coefficient (95% CI)	*P* -value	Coefficient (95% CI)	*P* -value	Coefficient (95% CI)	*P* -value
Intercept	27.65 (26.7-28.6)	<0.001	28.70 (27.7-29.7)	<0.001	27.39 (26.4-28.4)	<0.001	27.58 (26.6-28.6)	<0.001	27.94 (27.0-28.9)	<0.001
Geographic distance (km)	0.13 (0.1-0.2)	<0.001	0.11 (0.1-0.2)	<0.001	0.13 (0.1-0.2)	<0.001	0.11 (0.1-0.2)	<0.001	0.12 (0.1-0.2)	<0.001

Variable	Random sample6		Random sample7		Random sample8		Random sample9		Random sample10	
	Coefficient (95% CI)	*P* -value	Coefficient (95% CI)	*P* -value	Coefficient (95% CI)	*P* -value	Coefficient (95% CI)	*P* -value	Coefficient (95% CI)	*P* -value
Intercept	28.33 (27.4-29.3)	<0.001	28.14 (27.2-29.1)	<0.001	28.15 (27.2-29.1)	<0.001	28.45 (27.5-29.4)	<0.001	28.73 (27.8-29.7)	<0.001
Geographic distance (km)	0.11 (0.1-0.2)	<0.001	0.12 (0.1-0.2)	<0.001	0.10 (0.1-0.2)	<0.001	0.12 (0.1-0.2)	<0.001	0.11 (0.1-0.2)	<0.001
